# Quantitative histology analysis of the ovarian tumour microenvironment

**DOI:** 10.1038/srep16317

**Published:** 2015-11-17

**Authors:** Chunyan Lan, Andreas Heindl, Xin Huang, Shaoyan Xi, Susana Banerjee, Jihong Liu, Yinyin Yuan

**Affiliations:** 1 Department of Gynecologic Oncology, Sun Yat-sen University Cancer Centre, Guangzhou, China; 2 State Key Laboratory of Oncology in South China, Collaborative Innovation Centre for Cancer Medicine, Guangzhou, China; 3 Centre for Evolution and Cancer, The Institute of Cancer Research, London, UK; 4 Centre for Molecular Pathology, The Royal Marsden Hospital, London, UK; 5 Division of Molecular Pathology, The Institute of Cancer Research, London, UK; 6 Department of Pathology, Sun Yat-sen University Cancer Centre, Guangzhou, China; 7 Gynecology Unit, The Royal Marsden NHS Foundation Trust, London, UK

## Abstract

Concerted efforts in genomic studies examining RNA transcription and DNA methylation patterns have revealed profound insights in prognostic ovarian cancer subtypes. On the other hand, abundant histology slides have been generated to date, yet their uses remain very limited and largely qualitative. Our goal is to develop automated histology analysis as an alternative subtyping technology for ovarian cancer that is cost-efficient and does not rely on DNA quality. We developed an automated system for scoring primary tumour sections of 91 late-stage ovarian cancer to identify single cells. We demonstrated high accuracy of our system based on expert pathologists’ scores (cancer = 97.1%, stromal = 89.1%) as well as compared to immunohistochemistry scoring (correlation = 0.87). The percentage of stromal cells in all cells is significantly associated with poor overall survival after controlling for clinical parameters including debulking status and age (multivariate analysis p = 0.0021, HR = 2.54, CI = 1.40–4.60) and progression-free survival (multivariate analysis p = 0.022, HR = 1.75, CI = 1.09–2.82). We demonstrate how automated image analysis enables objective quantification of microenvironmental composition of ovarian tumours. Our analysis reveals a strong effect of the tumour microenvironment on ovarian cancer progression and highlights the potential of therapeutic interventions that target the stromal compartment or cancer-stroma signalling in the stroma-high, late-stage ovarian cancer subset.

Ovarian cancer is the leading cause of death in patients with gynaecological malignancies^1^. In most cases, the disease is diagnosed at an advanced stage and the mortality is high^2^. It remains a challenge to identify patients in whom treatment is likely to fail, and reliable biomarkers for predicting long-term survival at diagnosis are urgently needed. The tumour microenvironment is known to regulate cancer progression, evolution and development of treatment resistance in many cancer types^3^,^4^,^5^. In ovarian cancer, the influence of microenvironment has long been recognised^6^,^7^, and was further supported by recent studies where new subtypes closely related to the microenvironment were identified using molecular profiling data^8^,^9^,^10^. For example, gene expression data analysis has enabled the discovery of four subtypes in high-grade ovarian cancer with different clinical outcomes^8^. Of these, three were strongly associated with stromal and immune signatures. Tumours expressing immune signatures but not stromal signatures were found to be associated with a better prognosis than tumours expressing stromal signatures. More recently, a large consortium study, the Cancer Genome Atlas (TCGA), has described four high-grade serous ovarian cancer subtypes based on gene expression data, namely ‘immunoreactive’, ‘differentiated’, ‘proliferative’ and ‘mesenchymal’^9^. Subsequently, a follow-up study reported significantly different clinical outcome among these subtypes^10^. Again, samples with stronger immune response have the best overall survival^10^. The subtype with worst prognosis is the mesenchymal subtype, which presented higher amount of infiltrating stromal components such as myofibroblasts and microvascular pericytes with demoplastic stromal reaction^10^. Taken together, there is consistent evidence supporting active roles of the tumour microenvironment, especially in terms of immune and stromal infiltrate, in ovarian cancer progression.

While these molecular studies have changed the way we think of ovarian cancer heterogeneity, the translation of new knowledge into clinical advances has been limited by the cost and scale of molecular profiling. In addition, molecular diagnostic tests still present major challenges for many health care systems in the developed and developing world. Besides cost-associated issues, not all samples meet the standard for RNA quality and quantity required for such tests. Thus, alternative technologies that are more cost-effective and generally applicable could accelerate the translation of new research discovery and development of quantitative biomarkers for ovarian cancer.

In this paper, we explore the potential of automated image analysis for routine histology samples to enable quantitative analysis of the ovarian tumour microenvironment. While a large amount of efforts has been spent on molecular profiling of ovarian cancer, the use of automated image analysis to probe the complex tumour microenvironment in the spatial dimension has rarely been reported. The ability to objectively identify stromal components in ovarian tumour histology sections could enable the development of computer-assisted diagnosis, compliment molecular analysis, and lead to more effective therapeutics strategies by targeting the non-cancer components. Therefore, our aims are to 1) develop an accurate image analysis classifier for identifying heterogeneous cell types in ovarian tumour hematoxylin & eosin stained section, 2) systematically evaluate immune and stromal infiltration in these tumours, and 3) determine the clinical implication of microenvironmental heterogeneity in ovarian cancer.

## Materials and Methods

### Patient selection

After obtaining institutional review board approval, all patients with ovarian carcinoma at Sun Yat-sen University Cancer Centre who received primary surgery between May 1999 and December 2010 were reviewed. Patients with International Federation of Gynecology and Obstetrics (FIGO) stage III-IV disease who received primary surgical treatment were included. Clinical data including patient records, clinicopathologic characteristics, treatment regimen and follow-up data were collected. Informed consent was obtained from all patients. All methods were carried out in accordance with the approved guidelines of Sun Yat-sen University Cancer Centre.

### Patient characteristics

A total of 91 patients with FIGO stage III-IV ovarian cancer were identified and samples collected specifically from the ovary. Overall survival (OS) and progression-free survival (PFS) data were available for all 91 cases. Survival time was calculated from the date of surgery. OS was censored at the date of death or, for living patients, the date of last contact. PFS was censored at the date of first progression or death, whichever occurred first, or the date of last contact for the patients alive and without recurrent disease. Progression was defined as serially rising CA125 levels, or any clinical or radiographic evidence of new lesions as either local/regional relapse or distant metastasis. Median OS and PFS were 48.2 months (range 1.9–186.4 months) and 22 months (0.9–186.4), respectively. 71 (78.0%) patients experienced a recurrence that included both local and distant metastasis. Median age for these patients was 52 years (range, 22–82 years). Histology classification was based on the five main types: high-grade serous (HGSC), low-grade serous (LGSC), endometrioid, clear cell, mucinous^1^. All patients underwent debulking surgery followed by chemotherapy. The primary surgery mainly included total hysterectomy, salpingo-oophorectomy, omentectomy, nodal dissection, or bowel procedures. Optimal cytoreduction was considered as residual disease no larger than 1 cm in maximum diameter which was evaluated by surgeons at surgery^11^,^12^. 55 (60.4%) patients underwent optimal debulking, whereas 36 (39.6%) patients had suboptimal surgery. The majority of patients (87%) had 6–8 cycles of paclitaxel/docetaxel plus carboplatin/cisplatin postoperatively, and 13% of patients received adjuvant chemotherapy of cyclophosphamide, bleomycin plus carboplatin. All clinicopathologic characteristics of patients were summarised in Table 1.

### H&E image processing

Representative samples of primary ovarian cancer were obtained from formalin-fixed, paraffin-embedded (FFPE) tissue blocks in the Department of Pathology of Sun Yat-sen University Cancer Centre. 4 μm thick tissue sections were taken to perform H&E staining. H&E slides were scanned using an Aperio scanner (Aperio, San Diego, USA) at 20 x yielding digital images with a resolution of 0.5 μm/pixel. All acquired images were anonymised for research purpose such that no identifying information can be derived. Image analysis was performed with the R package CRImage and cell classification was performed using a training set of 900 cells provided by the pathologist and a Support Vector Machine^13^. For a separate testing set, single cell annotation was carried out for 615 cancer and 2,824 stromal cells but not for lymphocytes to avoid mis-annotation as lymphocytes often infiltrate to close contact with other cells. Instead, lymphocyte were scored in small images by their amount. 100 small images, each of 2,000 by 2,000 pixel or 1 mm by 1 mm, were randomly selected from the entire cohort. Of these, 87 contain lymphocytes and were used for validation.

### H&E pathological scores

Each of the H&E-stained whole-section slide images was evaluated by a pathologist and five areas were selected randomly under 4× magnification. Subsequently, tumour composition was assessed in these five areas under 8× magnification. Percentage of cancer cells and stromal infiltrate was scored and the average of the five selected areas was calculated. Pathological lymphocytic infiltration was scored as low, median, or high: low if there were low amount of lymphocytes, median if there was a light scattering of lymphocytes, and high if there was a prominent lymphocytic infiltrate.

### Immunohistochemical (IHC) validation

Thirteen representative formalin-fixed, paraffin-embedded sections were randomly selected. 4 μm thick tissue sections were dewaxed in xylene, and rehydrated through graded alcohol. After the inhibition of the endogenous peroxidase and an antigen retrieval process, the sections were incubated with primary antibodies anti-α smooth muscle actin (SMA; mouse monoclonal, 1:12,000 dilution, Sigma) for 50 minutes at 37 °C. Primary antibodies were detected by EnVision kit (K5007, DAKO). The reaction was visualised using 3,3-diaminobenzidine (DAB). Finally, the sections were counterstained with Mayer’s hematoxylin, dehydrated, and mounted. All thirteen sections were scored by an expert pathologist (DNR) as a percentage of SMA positive area per sample (Path SMA). Automated analysis of SMA-stained IHC images (Auto SMA) involved colour deconvolution^14^ to split blue hematoxylin and brown SMA stain. Dichotomisation of SMA image in positive and negative areas was achieved by employing Otsu thresholding^15^, a common image-processing approach to minimise intra-class variance.

### Statistical analysis

Statistical analysis was performed using R. Survival curves were generated using Kaplan-Meier method and difference in survival was tested using the log-rank test. Univariate and multivariate survival analysis were performed using the Cox proportional hazards model to identify prognostic factors. Effects were expressed as hazard ratios (HR) with 95% confidence intervals (CI). Statistical significance was defined as *P* < 0.05. Clinical pathologic associations with cell ratios were tested using Kruskal-Wallis test with continuous variable and Fisher’s exact test with categorical variable. Box plots represent median percentage and interquartile variation.

### Survival analysis with random sampling

We performed survival analysis with progressively decreasing amount of patient samples (100%, 99%, 98%, …, 60%) to test the reproducibility of our stromal cell ratio as a prognostic factor. Samples were randomly taken without replacement to test the effect of decreasing sample size on survival analysis and stratified into two equal-size groups based on stromal cell ratio, and univariate Cox analysis using OS data was performed. Log-rank test p-value was recorded. This procedure was repeated 1,000 times. The percentage of times where stromal cell ratio remained significant in univariate and multivariate models was calculated.

## Results

### Quantifying microenvironmental composition with image analysis of ovarian H&E tumour slides

**Figure 1:**
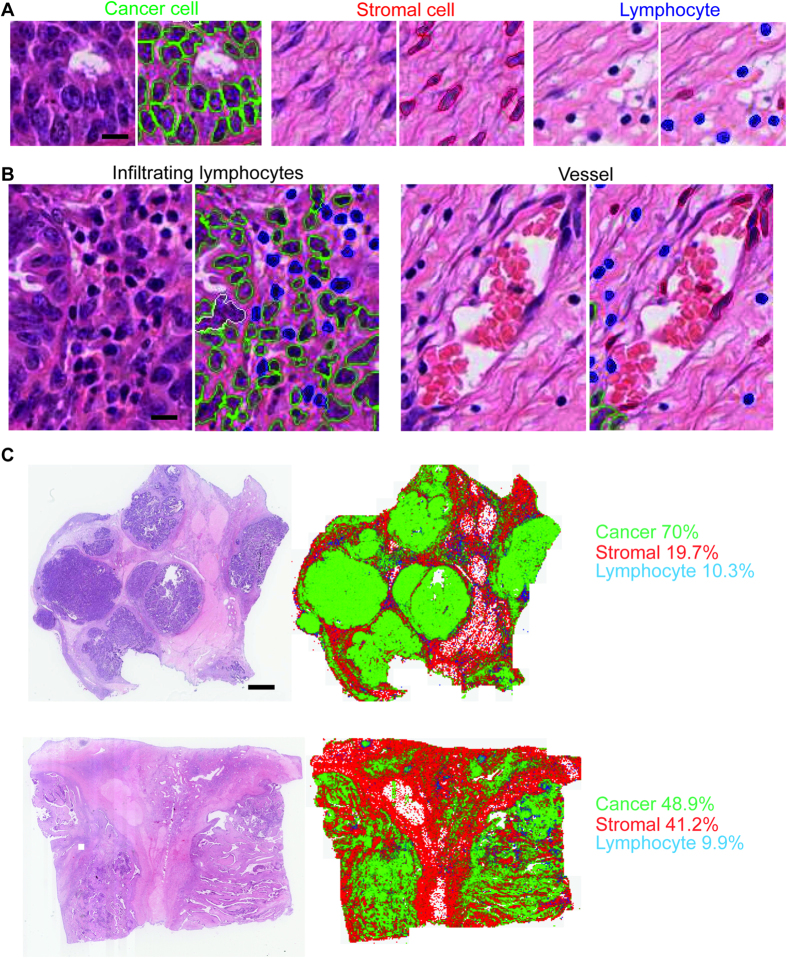
Image analysis for ovarian cancer histology sections. (**A**) Cell nuclei were classified into three cell classes based on morphology: epithelioid-like cancer cells (green), spindle-like stromal cells (red), round lymphocytes (blue) and artefacts (white). Scale bar: 12 μm. (**B**) Examples of infiltrating lymphocytes and vessel found in the sample. Scale bar: 12 μm. (**C**) Illustrative examples of two tumours to show the original H&E section and the spatial distribution and ratios of three cell types obtained from image analysis. Scale bar: 600 μm.

We developed a new image analysis pipeline for analysing H&E-stained whole tumour sections of primary ovarian cancer by designing a new cell classifier using a tool we previously developed^13^. This classifier was trained based on the annotation of 900 cells provided by an expert pathologist, from which 100 morphological features of every cell were quantified. It identifies cancer cells with epithelial morphology, stromal cells with spindle-like morphology which include fibroblasts and endothelial cells, and lymphocytes with small, round morphology (Fig. 1A,B). Using this classifier, all patient samples were analysed at single-cell resolution for the entire tumour sections (Fig. 1C). On average, 499,600 (interquartile range 255,700–642,900) cancer cells, 129,900 (72,960–171,200) stromal cells and 62,830 (33,240–84,820) lymphocytes were identified in each whole-tumour section.

**Figure 2:**
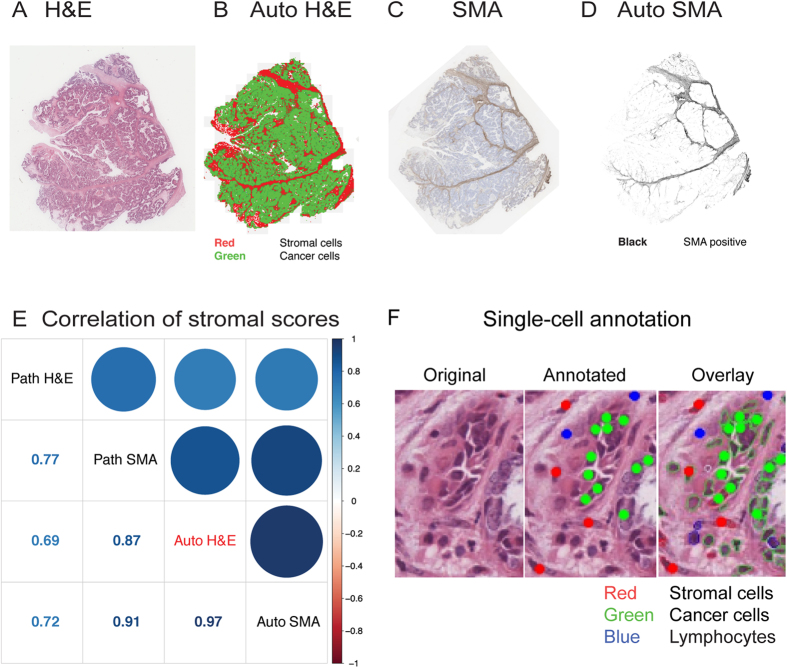
Validation of automated histology image analysis for ovarian cancer using three tests. (**A**) Original H&E section. (**B**) Automated scoring of H&E (Auto H&E) section depicting stromal cells in red and cancer cells in green. (**C**) SMA section. (**D**) Automated scoring of SMA (Auto SMA) section showing regions positive for the stain in black. (**E**) Correlation matrix comparing Auto H&E and Auto SMA scoring with pathological scores of H&E (Path H&E) and pathological scores of SMA (Path SMA). (**F**) Illustration of our analysis to compare annotation by hand and automated image analysis at single-cell resolution.

Subsequently, we performed three experiments to validate this new image analysis pipeline. Firstly, we demonstrated a high level of correlation between automated stromal cell identification based on H&E (Auto H&E; example in Fig. 2A,B) for both expert pathologist’s scores (Path SMA) as well as automated SMA scoring (Auto SMA; example in Fig. 2C,D) in IHC sections of 13 samples (Pearson correlation r = 0.87 with Path SMA and r = 0.97 with Auto SMA, Fig. 2E). Secondly, using a testing set of 3,439 cells manually annotated using our interactive platform in 61 randomly selected fields of view in three tumours (Fig. 2F), we showed that the classifier achieved an accuracy of 89.1% for stromal cells, 97.1% for cancer cells. For lymphocytes, since they are often in close contact with other cells and hard to annotate for single cells, we compared the amount of lymphocytes scored by image analysis and by the pathologist in 100 small images randomly selected from the entire cohort and observed a good level of correlation (p = 1.7 × 10^−7^).

### Microenvironment-derived subtypes based on image analysis were significantly associated with OS and PFS

**Figure 3:**
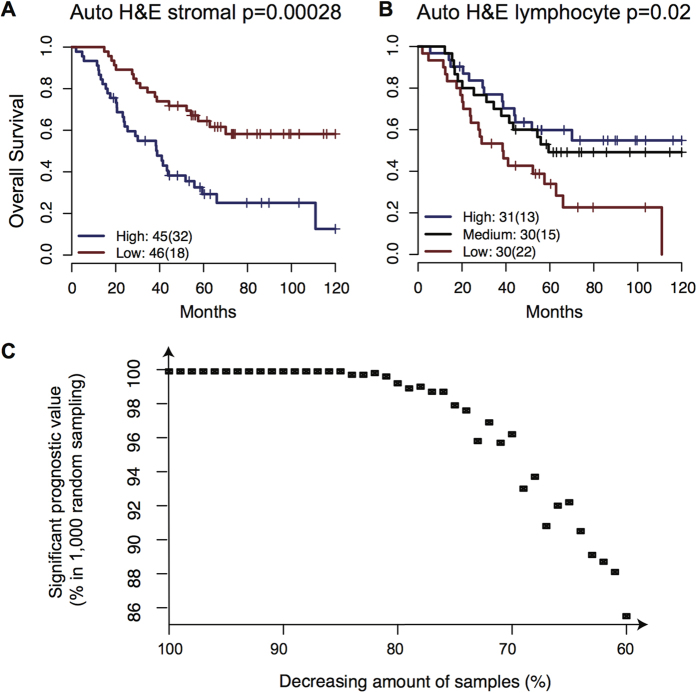
Quantitative microenvironmental composition measured by image analysis is significantly correlated with OS. Kaplan-Meier curves to demonstrate the differences in OS of patients divided into two equal-size groups according to (**A**). The ratio of stromal cells, and (**B**) three equal-size groups for lymphocyte ratio based on image analysis. The number outside brackets shows the number of patients in the group and the number inside the brackets shows the number of events. (**C**) Scatter plot to illustrate the percentage of times where stromal cell ratio remained significant in univariate survival analysis of OS as a function of sample size (60% to 100% of original cohort size) during 1,000 random sampling procedures.

Next, we tested the association between clinical outcome and microenvironmental composition provided by automated analysis and pathological scoring in ovarian cancer. To stratify patients according to automated image analysis results without making any assumption, we divided patients into two or three equal-size groups based on the amount of cell types. We observed that a high percentage of stromal cell correlates with significantly worse OS for both the two-group and three-group stratification (two-group stratification p = 0.00028, HR = 2.82, CI = 1.57–5.05, log-rank test; Fig. 3A, Supplementary Fig. 1A, Table 2) and PFS (two-group stratification p = 0.012, HR = 1.81, CI = 1.13–2.9; Supplementary Fig. 1B). On the other hand, a low lymphocyte ratio was associated with OS with the three-group but not the two-group stratification (Fig. 1B, Supplementary Fig. 1A). Patients with the lowest amount of lymphocyte ratio (lower 33 percentile) had significantly worst OS, but not PFS, compared to patients with higher lymphocyte ratio (lower 33% versus higher 67% OS p = 0.0058, HR = 0.46, CI = 0.26–0.81; Table 2, Fig. 3B). In addition, we observed a significant level of negative correlation between cancer and stromal ratio (Spearman correlation coefficient = −0.93) and a significant, positive correlation between cancer cell ratio and favourable prognosis (OS: p = 0.0025, HR = 0.42, CI = 0.24–0.75; PFS: p = 0.024, HR = 0.58, CI = 0.37–0.94), although it is not as strong as the prognostic value of stromal ratio. Compared with cell ratios as continuous variables, we found that the performance of stromal cell ratio and cancer cell ratio as continuous variables is comparable with, if not better than, their dichotomised equivalents (Supplementary Table 2). Lymphocyte ratio as a continuous variable on the other hand does not perform as well. For patient stratification, we henceforth used the 50% cutoff for stromal ratio and lower 33% cutoff for lymphocyte ratio to dichotomise these measures in subsequent analysis. The same trend of association between cell ratios and prognosis was observed when the same analysis was performed in the serous samples only (82 samples), thus the prognostic association was not due to histology subtype heterogeneity (Supplementary Fig. 2).

To test the reproducibility of stromal cell ratio in patient subsets, we performed random sampling with progressively less samples and showed that even with only 60% of samples (54 patients), 86% of the times in 1,000 random sampling procedures stromal cell ratio remained significant in univariate analysis of OS (Fig. 3C). With 80% or more samples, >99% of the times stromal cell ratio was significant, supporting the reproducibility of prognostic value of stromal cell ratio within our cohort.

### Comparison of image analysis with clinicopathologic scores

Next, we tested prognostic value of clinicopathologic scores. We found that pathological scores of microenvironmental composition were not associated with overall survival (OS) or progression-free survival (PFS) (p > 0.1 for two or three-group stratification, Supplementary Fig. 3). On the other hand, known prognostic factors for ovarian cancer including debulking status were significantly associated with OS, while stage and histology type were not (Supplementary Fig. 4A, Table 2). Dichotomised age groups (>60 versus ≤60) showed a more significant prognostic association than age as a continuous variable (OS log-rank test p = 0.0024 versus p = 0.021), thus age groups were used in our analysis. The lack of association between prognosis and well-known prognostic factors such as stage could be explained by the fact that the majority of patients in this cohort presented advanced stage, high-grade serous cancers (83% stage III and 89% serous; Table 1). In terms of PFS, we found that only debulking status and age were significantly correlated with poor prognosis (debulking: p = 0.0027, HR = 2.05, 95% CI = 1.27–3.32; age p = 0.015, HR = 2.06, CI = 1.14–3.75) (Supplementary Fig. 4B, Table 2). Treatment heterogeneity was not associated with PFS (p = 0.22) or OS (p = 0.71).

### Association of microenvironmental composition with clinicopathologic characteristics

We examined the association between stromal and lymphocyte ratio and all clinicopathologic variables. The only associations between cell ratios and clinicopathologic variables were stromal ratio with debulking status, and lymphocyte ratio with histology type (p ≤ 0.05, kruskal test). Stromal cell ratio is higher in suboptimal compared with optimal debulking status with borderline significance (p = 0.051, Kruskal test). Lymphocyte ratio differed significantly among histology subtypes (p = 0.019, Kruskal test), mainly due to its lower level in low-grade serous subtype than the other subtypes (p = 0.016, Kruskal test). Stromal cell ratio is higher in high-grade tumours (p = 0.028) whilst lymphocyte ratio is significantly lower in these tumours (p = 0.0048).

### Microenvironment-derived subtypes were prognostic independent of clinical parameters

**Figure 4:**
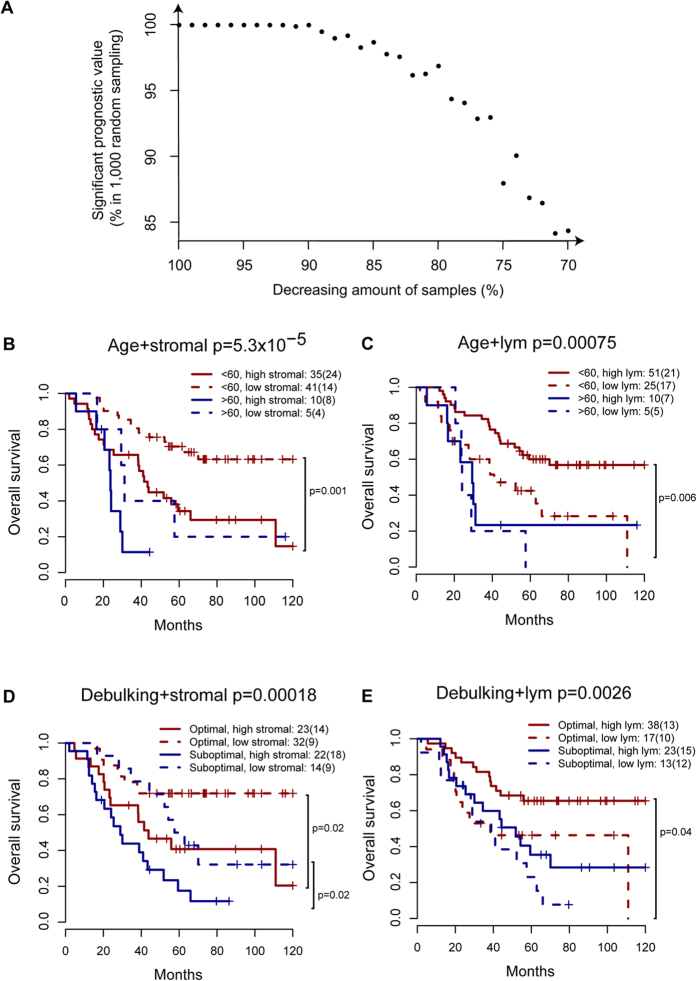
Multivariate analysis combining microenvironmental composition with clinical parameters. (**A**) Scatter plot to illustrate the percentage of times where stromal cell ratio was significant in multivariate Cox analysis of OS as a function of sample size from 70% to 100% of original cohort size (91 patients) during 1,000 random sampling procedures. (**B**–**E**) Kaplan-Meier curves illustrating differences in survival according to stromal or lymphocyte ratio and age or debulking status. Log-rank test p-values to show significant differences between groups were marked.

To evaluate whether microenvironmental composition adds useful information to existing subtype stratification of ovarian cancer, we performed multivariate analysis to include all parameters that were prognostic in univariate analysis, that is, debulking status and age. Stromal cell ratio was strongly prognostic in multivariate models for both PFS and OS (Table 2). Random sampling was again performed for stromal cell ratio in multivariate analysis of OS. We found that with more than 74% (67) samples, >90% of the times stromal cell ratio was found to remain significant; and with more than 87% (79) samples, >99% of the times stromal cell ratio was significant in multivariate analysis (Fig. 4A). Lymphocyte ratio was found to be significant in multivariate analysis for OS but not PFS (Table 2). While debulking status was significant in the multivariate analysis of PFS, age is the only clinical parameters to remain significant in the multivariate analysis of OS (Table 2).

Thus, we combined microenvironmental composition measures with these two parameters to define new patient groups. The majority of cases were under age of 60 at surgery (83%). Stromal cell and lymphocyte ratio were used to stratify patients of age <60 into two groups with distinctly different OS (Fig. 4B,C, stromal p = 0.001, HR = 2.88, CI = 1.48–5.59; lymphocyte p = 0.006, HR = 0.42, CI = 0.22–0.8). Specifically, higher stromal cell ratio and age <60 together defines a group of patients with 63% of OS probability 10 years after surgery, an outlook rare for high-grade ovarian cancer (Fig. 4B). In addition, stromal cell ratio further stratified patients with different debulking status (stromal low and high in: optimal debulking p = 0.018, HR = 2.6, CI = 1.14–6.12; suboptimal debulking p = 0.019, HR = 2.55, CI = 1.13–5.77; Fig. 4D). Lymphocyte ratio, on the other hand, could only stratify patients with optimal debulking (p = 0.037, HR = 0.41, CI = 0.18–0.94) but not suboptimal (p = 0.17; Fig. 4E). Difference in PFS according to combined variables, on the other hand, were not as prominent (Supplementary Fig. 5).

### Lymphocyte and stromal cell ratio co-defined a prognosis classifier

**Figure 5:**
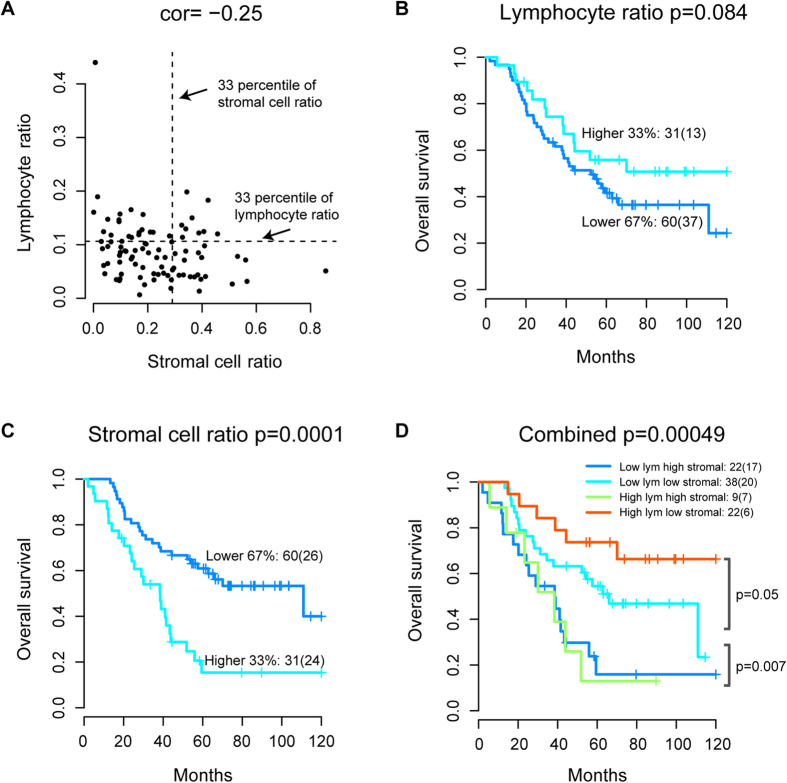
Combining lymphocyte and stromal cell ratio for an integrated microenvironment-based classifier. (**A**) Scatter plot to show correlation between lymphocyte and stromal cell ratio; dashed lines marked the higher 33 percentile for both measures, which will be used to dichotomised the measures for subsequent analysis. (**B**) Kaplan-Meier curves to demonstrate differences in OS according to lymphocyte ratio. (**C**) Kaplan-Meier curves to demonstrate differences in OS according to stromal cell ratio. (**D**) Kaplan-Meier curves to demonstrate differences in OS according to a combined analysis of lymphocyte and stromal cell ratio. P-values denote significance in differences between the high lymphocyte low stromal and low lymphocyte low stromal groups; and between the low lymphocyte low stromal and high stromal groups.

Given the prognostic value of lymphocyte and stromal ratio in ovarian cancer, we next asked if these parameters can co-define a better prognostic classifier. There was a weak negative correlation between them (Spearman correlation coefficient −0.25, p = 0.016, Fig. 5A). We selected one-third of the patients with the highest lymphocyte ratio as the immunoreactive group. This group of patients has higher 10-year OS probability than the rest of the patients (54% versus 23%), although the difference in OS is not significant (p = 0.084, log-rank test, Fig. 5B). Similarly, we also defined one-third of patients with the highest stromal ratio. These patients have significantly worse OS compared with the rest of patients (p = 9.9 × 10^−5^, HR = 2.92, CI = 1.66–5.15, log-rank test, Fig. 5C).

Notably, when patients were assigned to groups based on both criteria, we observed that the group of patients with high lymphocyte and low stromal ratio has a 10-year OS probability of 70%, compared with the 22% for all the other patients (p = 0.003, HR = 0.29, CI = 0.12–0.7, log-rank test, Fig. 5D). In contrast, patients with high stromal ratio, regardless of their lymphocyte ratio, have the worst prognosis, with 15% of OS probability at 5 year after surgery (p = 9.9 × 10^−5^, HR = 2.92, CI = 1.66–5.15). Overall, a three-group classifier, consisting of a high lymphocyte low stromal (low risk), a low lymphocyte low stromal (medium risk), and a high stromal (high risk) group, is a significantly prognostic factor (p = 0.0001, HR = 0.55, CI = 0.4–0.76). This classifier was the most significant variable in univariate analysis for both OS and PFS, but similar in performance with stromal cell ratio in multivariate analysis (Table 2). Examination by an expert pathologist in cases with high lymphocyte ratio and high stromal cell ratio confirmed extensive lymphocyte infiltrate in the former and spindle-like endothelial cells and fibroblasts in the latter.

## Discussion

We presented a quantitative analysis of microenvironmental composition in ovarian cancer of advanced stage (FIGO stage III and IV) using H&E-stained histology samples. There is increasing evidence to support the importance of tumour microenvironment in ovarian cancer progression and subtype definition^7^,^10^. However, the majority of quantitative studies were performed using molecular profiling data using microarrays or sequencing technology which requires high quality RNA^9^,^10^,^16^. Our approach offers a new, cost-effective opportunity to objectively compare samples routinely generated in pathology to study the microenvironment and to identify high-risk patients of ovarian cancer. Our focused study on ovarian tumour microenvironment is thus different from others that apply histology image analysis in ovarian cancer^17^,^18^, since we consider both immune and stromal infiltration at single-cell resolution.

The key finding in our study is that high amount of stromal cells in ovarian cancer was significantly associated with poor prognosis. A classifier based on stromal ratio in the tumours can effectively separate patients into equal-size groups with distinctly different survival profiles, independent and complementary to known clinical variables. This observation is consistent with the seminal study by Tothill *et al.*, in which a reactive stroma gene expression subtype was identified with the worst prognosis in ovarian cancer^8^, but our data highlighted the striking prognostic value of stromal infiltrate quantified with a new automated histology analysis approach using the most common, basic histology staining, H&E. In their study, pathological assessment of H&E slides of cases in the reactive stroma subtypes revealed higher amount of desmoplasia that is associated with infiltrating stromal cells^8^. Genes differentially expressed in the stromal subtype compared to other subtypes include markers of active myofibroblasts, endothelial cells and pericytes^8^, which is consistent with our image analysis cell classification scheme. In our study, pathological review confirmed that the stromal cells identified by image analysis encompass stroma fibroblasts, myofibroblasts, fibrocytes, vascular pericytes and endothelial cells. Although we currently do not differentiate these cell types, future studies to identify these cells and their individual contributions to the aggressive cancer phenotype could be key to new therapeutic targets for advanced ovarian cancer.

In addition, the amount of lymphocytic infiltration was found to correlate with favourable prognosis. T-cell infiltrate has been shown to be of clinical interest in ovarian cancer^19^. An immunoreactive subtype was consistently identified in multiple molecular studies, where extensive T-cell infiltration in these cases was also observed in histology review^9^,^10^,^16^. For example, a subtype with high immune signature identified in Tothill *et al.* was presented with extensive CD3 + cell infiltration and better prognosis compared to other patients^8^. In contrast, low number of CD3 + T-cells were found in the stromal reactive subtype^8^. This negative association between T-cell infiltrate and stromal reactive trait was also identified in our study, where a negative correlation was found between lymphocytic infiltration and stromal cell ratios. Taken together, we postulate that we may have identified the morphological counterparts of these molecular subtypes. However, this needs to be verified using datasets where both histology and genomic data are available such as the TCGA^9^.

Limitations in our study include its retrospective nature, heterogeneous treatment regimen, mixed histology type, sample size and the lack of an independent test set. Nevertheless, we did not find an association between treatment regimen and prognosis in our cohort. By simply dividing patients into equal-size groups according to our new measures without the need for selecting a cutoff, significant differences in survival were observed. Together with the resampling experiments, our data suggests that the histology image-based measures could be intrinsic and clinically relevant features of late-stage ovarian cancer. Since our focus is late stage cancers, our cohort contained a mixture of histology types and the proposed prognostic factors were reproducible in high-grade serous cancers, the majority of our cohort. Our new cell classifier achieved a good degree of accuracy compared with pathological scores of H&E (Auto H&E versus Path H&E r = 0.69) and SMA-stained sections (Auto H&E versus Path SMA r = 0.87). The lower degree of correlation with pathological scores in H&E was likely due to the fact that our image analysis scored the entire tumour whilst pathological review was based on selected fields, which is a common practice. Although prognostic value of automated scores far exceeded that of pathological scores, pathological expertise was essential to the success of this study for training the cell classifier. We thus envision an efficient medical system where pathologists train histology analysis tools to perform repetitive tasks such as cell counting with minimal bias.

Cancer development depends on the recruitment and evasion of host cells^20^,^21^. For this reason, the tumour microenvironment is an attractive target for developing new therapeutic strategies that will complement current treatment regimen^3^,^5^. On-going clinical trials have already demonstrated initial success in applying microenvironment-targeting treatment, including immunotherapy, to a number of cancer types^22^,^23^,^24^. Development of these strategies in ovarian cancer will likely transform the way ovarian cancer patients will be treated in the future. Taken together, our study presents an opportunity to enable large-scale studies of ovarian tumour microenvironment using the enormous amount of routine histology samples generated to date. Such efforts will facilitate the development of a computer-assisted diagnosis tool for predicting likely outcome for a patient and identifying high-risk patients for intense monitoring, and further new treatment strategies involving microenvironment-targeting drugs.

### Data and software availability

R code and the full dataset for performing the proposed methodology and reproducing reported results are provided as Sweave files and supplementary data on www.yuanlab.org for full reproducibility.

## Additional Information

**How to cite this article**: Lan, C. *et al.* Quantitative histology analysis of the ovarian tumour microenvironment. *Sci. Rep.* **5**, 16317; doi: 10.1038/srep16317 (2015).

## Supplementary Material

Supplementary Information

Supplementary Table 1

Supplementary Table 2

## Figures and Tables

**Table 1:** Patient clinicopathologic characteristics according to stromal cell and lymphocyte ratio.

Factor	Stromal high	Stromal low	P	Lymphocytehigh	Lymphocytelow	P
Number	45	46		61	30	
Follow-up						
Median	38.3	62.2		55.8	35.9	
Range	2–186.4	14.8–155.2		5.5–186.4	2–111	
Age			0.76			0.71
Median	55	52		52	53	
Range	(22–75)	(36–82)		(22–82)	(27–68)	
Death			0.0031*			0.015*
No	13 (28.9%)	28 (60.9%)		33 (54.1%)	8 (26.7%)	
Yes	32 (71.1%)	18 (39.1%)		28 (45.9%)	22 (73.3%)	
Recurrence			0.21			0.43
Yes	38 (84.4%)	33 (71.7%)		46 (75.4%)	25 (83.3%)	
No	7 (15.6%)	13 (28.3%)		15 (24.6%)	5 (16.7%)	
Histology			0.056			0.034*
High-grade serous carcinoma	37 (82.2%)	40 (87%)	0.57	53 (86.9%)	24 (80%)	0.54
Low-grade serous carcinoma	5 (11.1%)	0 (0%)	0.026*	1 (1.6%)	4 (13.3%)	0.039*
Mucinous	2 (4.4%)	2 (4.3%)	1	2 (3.3%)	2 (6.7%)	0.6
Endometrioid	1 (2.2%)	4 (8.7%)	0.36	5 (8.2%)	0 (0%)	0.17
FIGO stage			0.44			0.93
IIIa	0 (0%)	1 (2.2%)	1	1 (1.6%)	0 (0%)	1
IIIb	1 (2.2%)	4 (8.7%)	0.36	4 (6.6%)	1 (3.3%)	1
IIIc	39 (86.7%)	37 (80.4%)	0.57	50 (82%)	26 (86.7%)	0.77
IV	5 (11.1%)	4 (8.7%)	0.74	6 (9.8%)	3 (10%)	1
Debulking			0.088			0.65
Optimal	23 (51.1%)	32 (69.6%)		38 (62.3%)	17 (56.7%)	
Suboptimal	22 (48.9%)	14 (30.4%)		23 (37.7%)	13 (43.3%)	
Primary chemotherapy regimen			0.55			0.74
TC/TP†	38 (84.4%)	41 (89.1%)		52 (85.2%)	27 (90%)	
CBP‡	7 (15.6%)	5 (10.9%)		9 (14.8%)	3 (10%)	

^†^TC/TP: Paclitaxel/docetaxel plus carboplatin/cisplatin.

^‡^CBP: Cyclophosphamide, bleomycin plus carboplatin.

**Table 2:** Prognostic value of stromal cell ratio, lymphocyte ratio, cancer cell ratio, and stromal-lymphocyte combined in ovarian cancer using progression-free and overall survival.

Type	Variable	PFS			OS		
		_HR (CI)_	_*p*_	_Conc_	_HR (CI)_	_*p*_	_Conc_
Uni	Debulking	2.05 (1.27–3.32)	0.0027	0.566	2.2 (1.26–3.84)	0.0046	0.581
	Age	2.06 (1.14–3.75)	0.015	0.55	2.82 (1.44–5.5)	0.0015	0.573
Uni	*Stromal*	1.81 (1.13–2.9)	0.012	0.59	2.82 (1.57–5.05)	0.00028	0.63
Multi	*Stromal*	1.75 (1.09–2.82)	0.022	0.62	2.54 (1.40–4.60)	0.0021	0.69
	Debulking	1.87 (1.14–3.05)	0.013		1.79 (1.01–3.14)	0.047	
	Age	1.80 (0.98–3.31)	0.059		0.02 (1.15–4.60)	0.018	
Uni	*Lymphocyte*	0.75 (0.46–1.22)	0.24	0.538	0.46 (0.26–0.81)	0.0058	0.581
Multi	*Lymphocyte*	0.86 (0.52–1.42)	0.562	0.61	0.53 (0.30–0.93)	0.027	0.67
	Debulking	1.88 (1.15–3.08)	0.012		1.86 (1.04–3.30)	0.0351	
	Age	1.88 (1.15–3.08)	0.059		2.33 (1.18–4.61)	0.0147	
Uni	*Cancer*	0.58 (0.37–0.94)	0.024	0.591	0.42 (0.24–0.75)	0.0025	0.62
Multi	*Cancer*	0.59 (0.36–0.94)	0.028	0.623	0.46 (0.26–0.82)	0.0086	0.693
	Debulking	1.88 (1.15–3.07)	0.012		1.84 (1.03–3.28)	0.039	
	Age	1.86 (1.01–3.42)	0.048		2.39 (1.20–4.8)	0.04	
Uni	*Combined*	0.72 (0.57–0.91)	0.0058	0.61	0.56 (0.41–0.77)	0.00015	0.65
Multi	*Combined*	0.76 (0.6–0.96)	0.024	0.63	0.60 (0.44–0.81)	0.0012	0.71
	Debulking	1.77 (1.04–2.82)	0.035		1.76 (0.97–3.17)	0.06	
	Age	1.76 (0.96–3.26)	0.069		2.17 (1.08–4.37)	0.031	

Cancer and stromal cell ratio were dichotomised into two groups of equal size, and lymphocyte ratio were dichotomised into a lower 33% and a higher 67% group. Combined: patient groups consisting of a high stromal, a low stromal high immune, and a low stromal low immune group. Uni: Univariate Cox regression; Multi: Multivariate Cox regression; Conc: Concordance; HR: Hazard Ratio; CI: Confidence Interval. *p < 0.05; **p < 0.01.
