# Supplementary material for: Quantitative histology analysis of the ovarian tumour microenvironment
Source: Sci Rep. 2015 Nov 17;5:16317. doi: 10.1038/srep16317 (PMC4647219; doi:10.1038/srep16317)
Supplement: Supplementary Information [file srep16317-s1.pdf]

<u>**Supplementary Information**</u>

# Quantitative histology analysis of the ovarian tumour microenvironment


Chunyan Lan[1,2#], Andreas Heindl[3,4,5#], Xin Huang[1,2#], Shaoyan Xi[2,6], Susana Banerjee[7], Jihong Liu[1,2*], Yinyin Yuan[3,4,5*]

[1] Department of Gynecologic Oncology, Sun Yat-sen University Cancer Centre, Guangzhou, China

[2] State Key Laboratory of Oncology in South China, Collaborative Innovation Centre for Cancer Medicine, Guangzhou, China

[3] Centre for Evolution and Cancer, The Institute of Cancer Research, London, UK

[4] Centre for Molecular Pathology, The Royal Marsden Hospital, London, UK

[5] Division of Molecular Pathology, The Institute of Cancer Research, London, UK

[6] Department of Pathology, Sun Yat-sen University Cancer Centre, Guangzhou, China

[7] Gynecology Unit, The Royal Marsden NHS Foundation Trust, London, UK

*Corresponding authors:

Yinyin Yuan, Ph.D., The Institute of Cancer Research, London, UK. Tel: +44-20-89156632. Email: Yinyin.Yuan@icr.ac.uk.

Jihong Liu, MD, PhD, Sun Yat-sen University Cancer Centre, Guangzhou, China. Tel: +86-20-87343102. Email: liujih@mail.sysu.edu.cn.

[#] equal contributions.

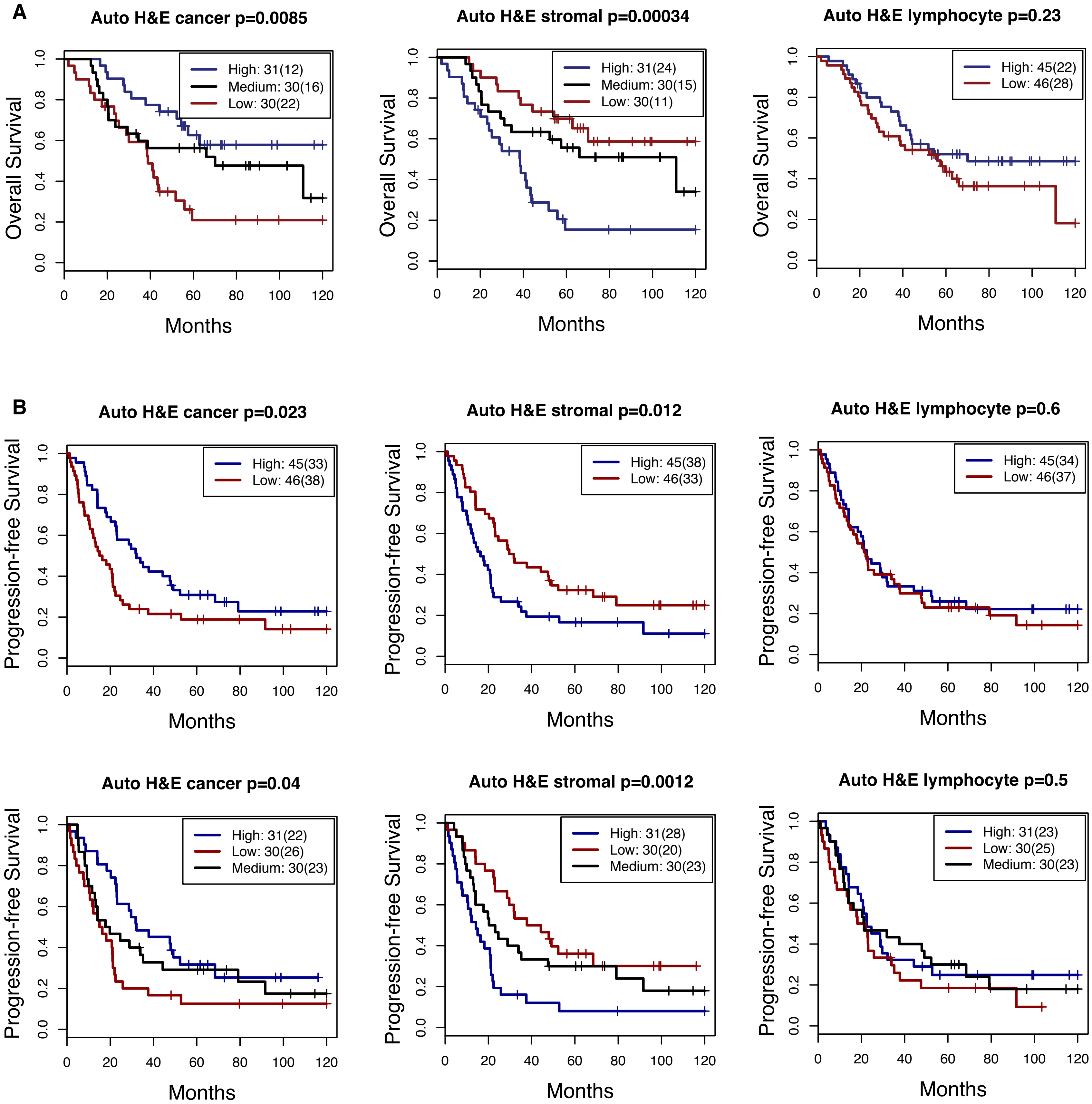


**Supplementary Figure 1.** Kaplan-Meier curves to demonstrate the differences in A. OS and B. PFS according to image analysis results of cell ratios by dividing patients into two groups of equal sizes or three equal-size groups.

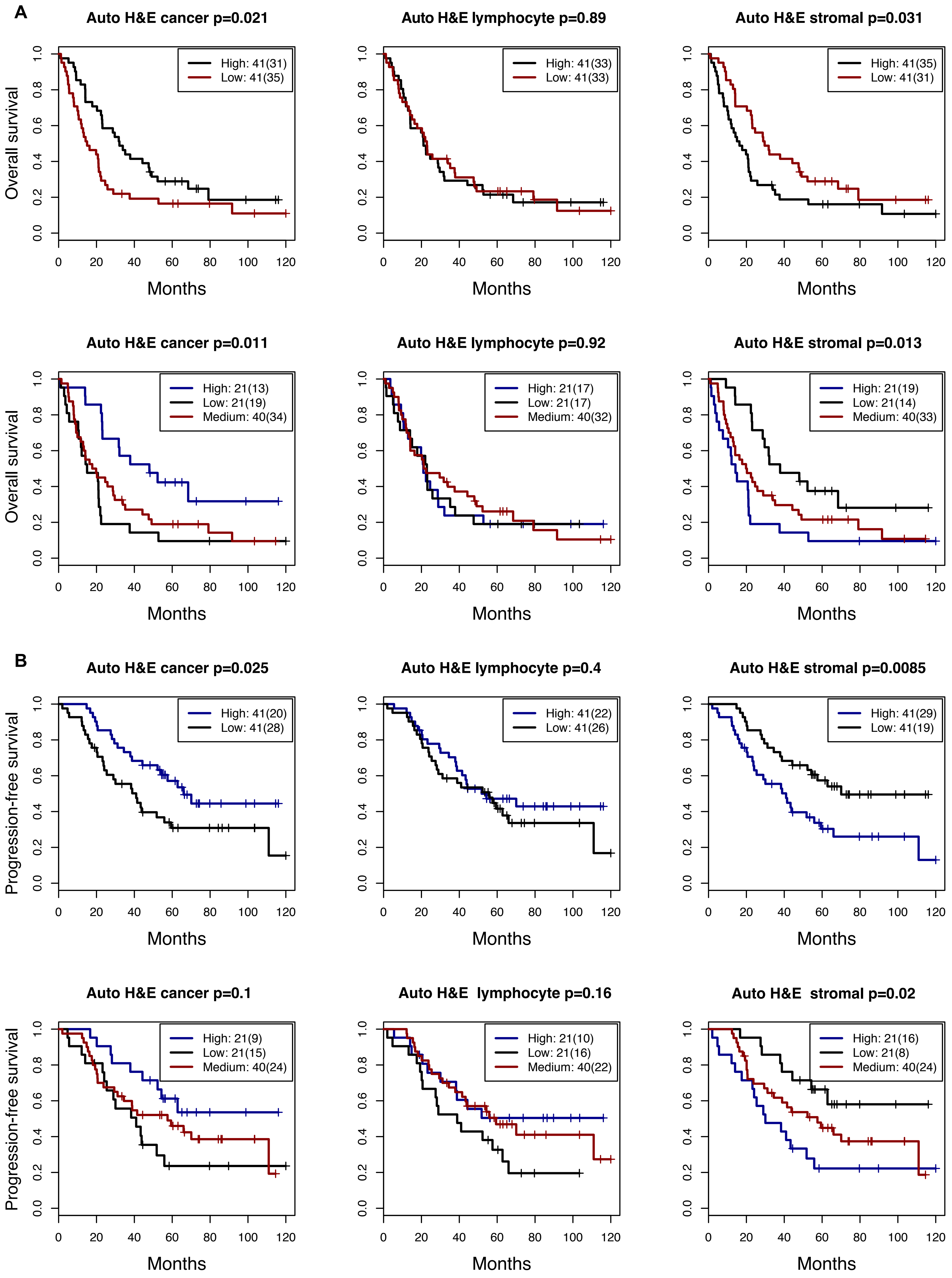


**Supplementary Figure 2.** Kaplan-Meier curves to demonstrate the differences in A. OS and B. PFS according to image analysis results of cell ratios in high-grade serous histology type only by dividing patients into two groups of equal sizes or three equal-size groups.

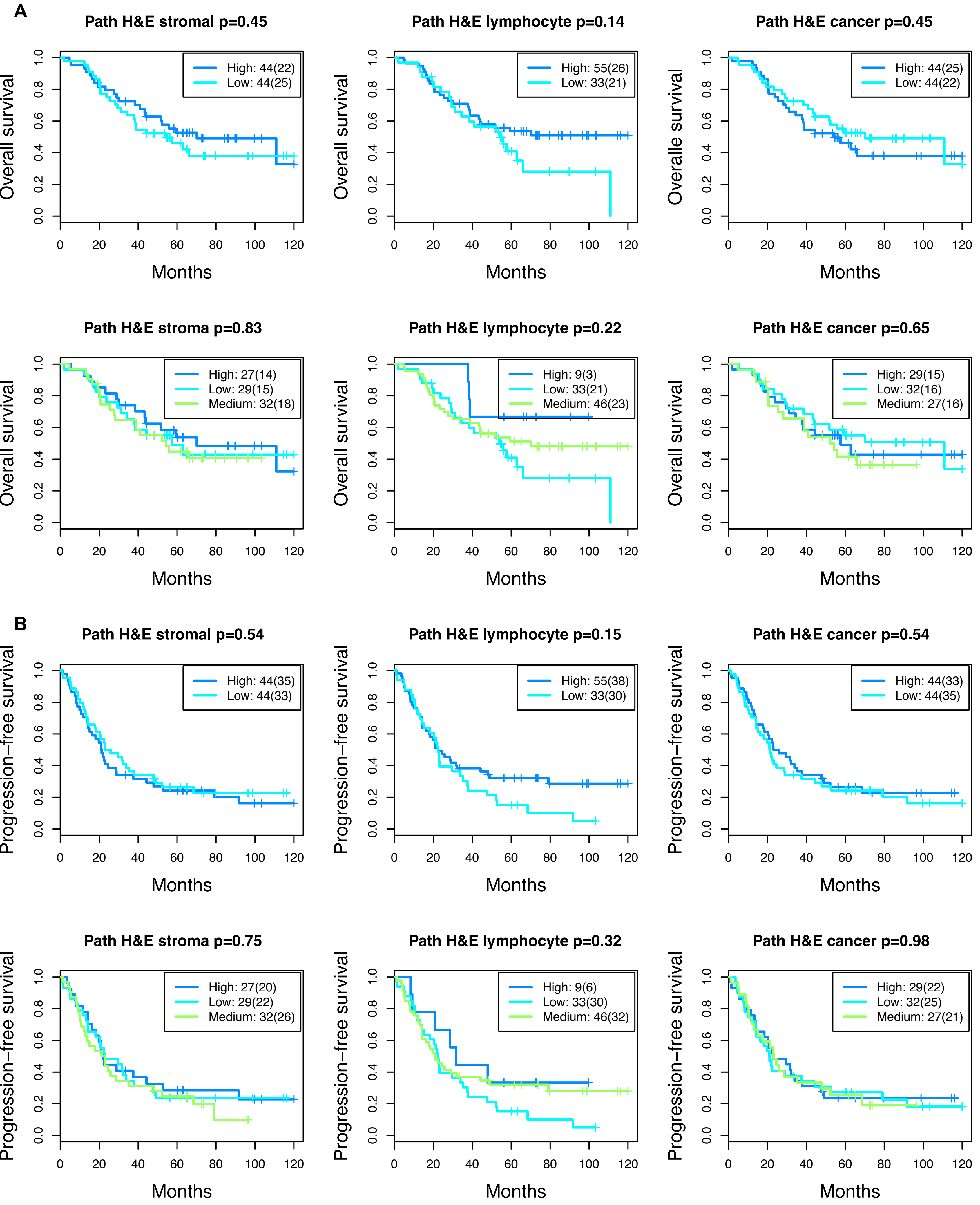


**Supplementary Figure 3.** Kaplan-Meier curves to illustrate the association between pathological scores of tumour composition and A. OS, B. PFS of ovarian cancer. Kaplan-Meier curves to demonstrate the differences in survival according to pathologists' scores of cancer and stromal cell ratios grouped into two or three equal-size groups. Lymphocytes were scored as "low", "medium" and "high". Due to the small size of the "high" group also a two-group stratification by merging the "medium" and "high" group is provided. The number outside brackets shows the number of patients in the group and the number inside the brackets shows the number of events.

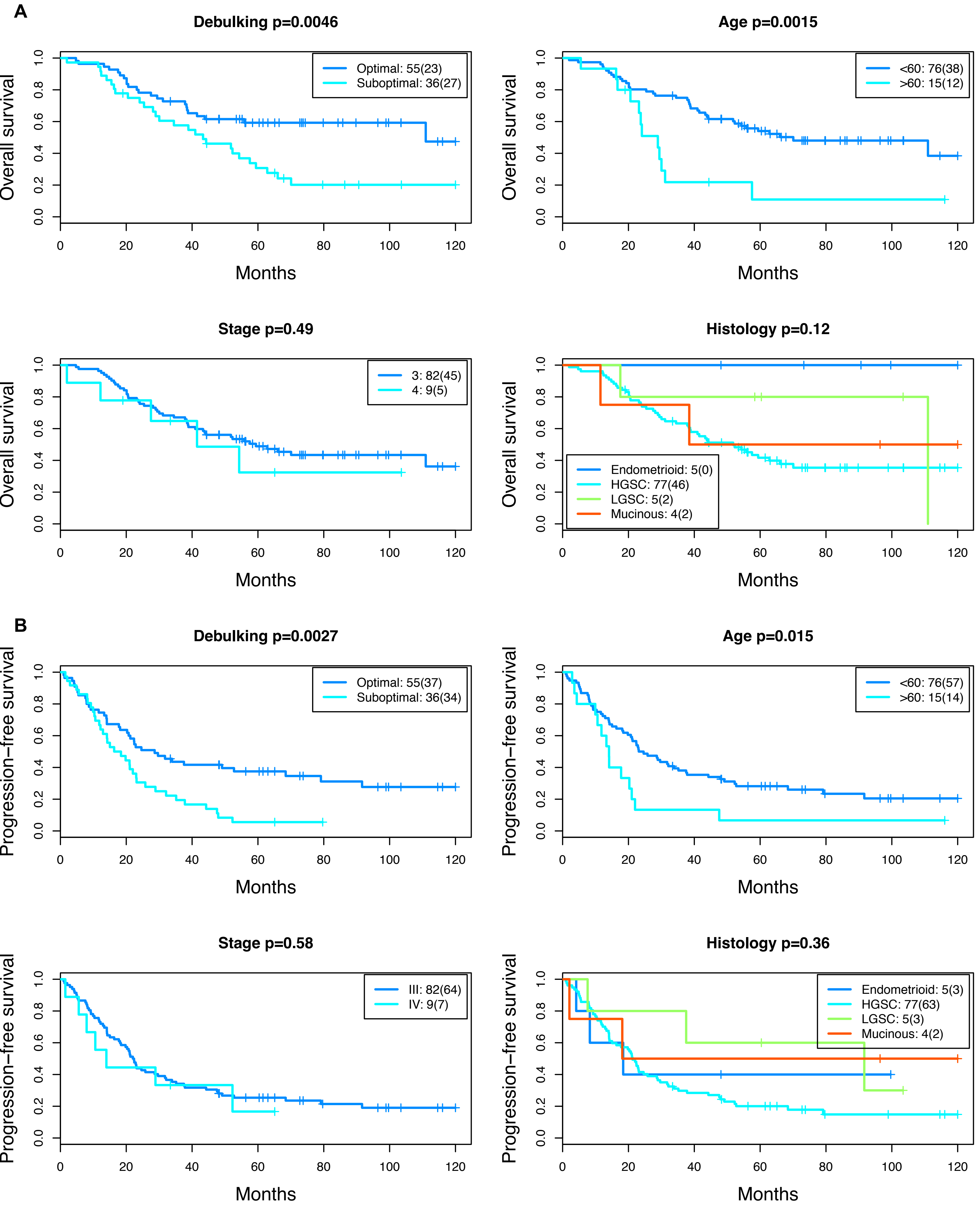


**Supplementary Figure 4.** Kaplan-Meier curves to illustrate the association between clinical variables and A. OS, B. PFS of ovarian cancer. These include debulking status, age, stage and histology type.

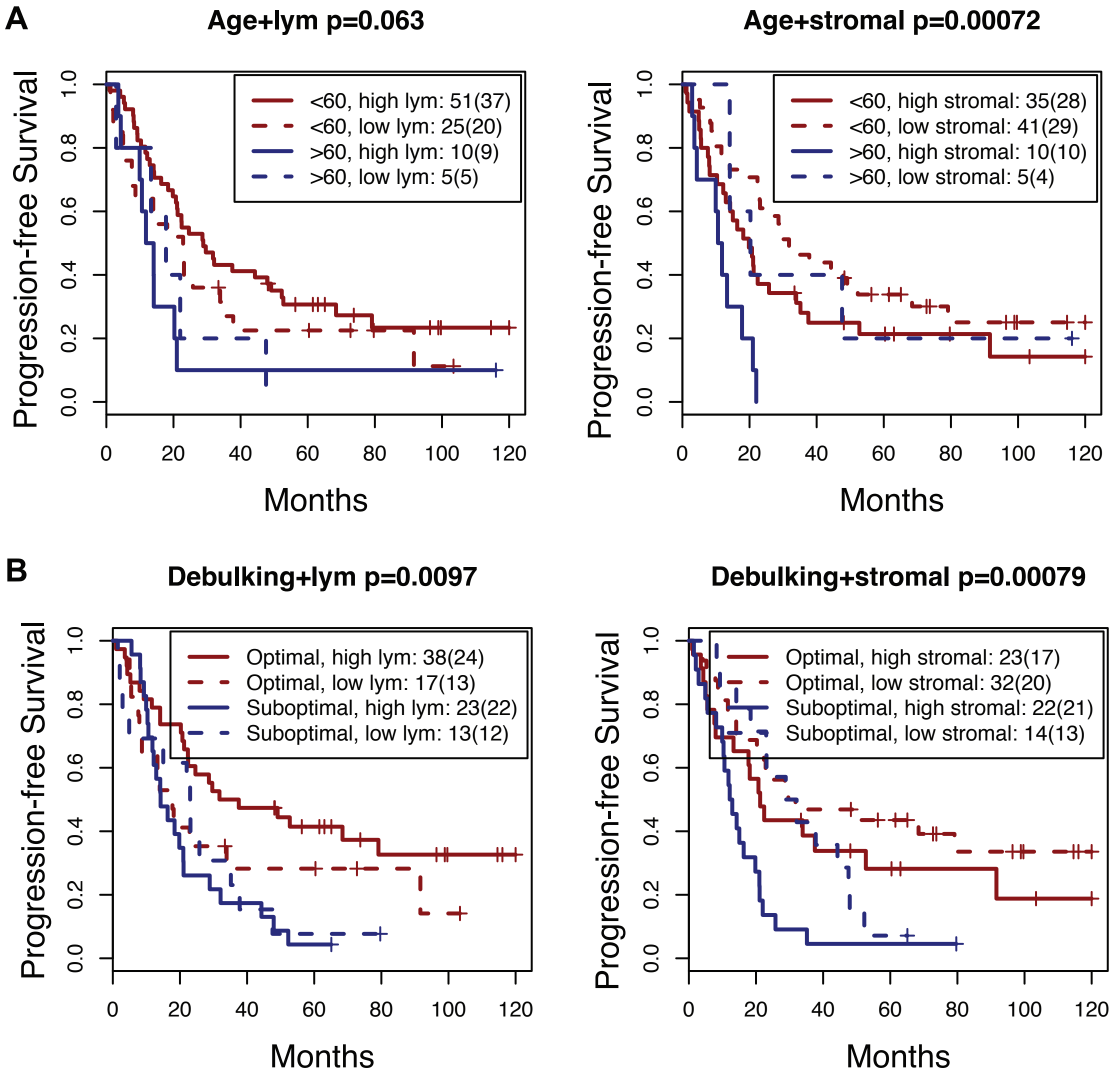


**Supplementary Figure 5.** Kaplan-Meier curves to demonstrate the differences in PFS according to combined microenvironmental composition and clinical parameters that were significant in multivariate analysis.

# R code and data for – Quantitative histology image analysis for Ovarian tumor microenvironment –

## Contents


**1 Preparation** **2**
- 1.1 Load data and functions . . . . . 2
- 1.2 Clinical parameters of the ovarian cancer samples . . . . . 2

**2 Distribution** **4**

**3 Prognostic value of the microenvironment** **8**
- 3.1 Pathological scores . . . . . 8
- 3.2 Clinical parameters . . . . . 10
- 3.3 Stromal, lym and cancer on PFS . . . . . 12
- 3.4 Stromal, lym and cancer on OS . . . . . 15

**4 Combining factors** **21**
- 4.1 Clinical and cell ratio . . . . . 21
- 4.2 Lym and stroma ratio . . . . . 24

**5 Only serious cases** **28**

**6 Sampling analysis** **31**
- 6.1 Univariate analysis . . . . . 31
- 6.2 Multivariate analysis . . . . . 31

**7 Session Info** **33**

# 1 Preparation

## 1.1 Load data and functions

To reproduce our result, first source the R functions needed for analysis and plotting and load the data for the 91 ovarian cancer samples, which is available as part of the paper supplement.

```
library(survival)
load("./data/trait.rdata")
source("functions.R")
```

## 1.2 Clinical parameters of the ovarian cancer samples

Each row in this clinical file is a sample/patient. Columns indicate: Chemotherapy regimen, age, degulking status, staging, grade, two tier type, histology type, recurrence, outcome, number of cancer, stromal, lymphocytes (nTumour, nOthers, nLymphocyte), proportions of lymphocyte, cancer, and stromal cell (lym, cancer, stromal), pathological scores of cancer, stromal, lymphocyte ratio (canerPath, stromalPath, lymPath), 10-year progression-free survival (S_10year), overallsurvival (OS_10year), histology type.

```
# Show clinical information data
head(trait)

##   Slide.No. Chemotherapy.regimen..1.TC.TP..2.CBP age.years.
## 1    269004                                    2         57
## 2    272077                                    2         48
## 3    274226                                    2         31
## 4    274908                                    2         45
## 5    285207                                    2         67
## 6    341026                                    1         46
##   age.for.calculation.1._60.years..2._60.years
## 1                                            1
## 2                                            1
## 3                                            1
## 4                                            1
## 5                                            2
## 6                                            1
##   debulking.status..1.optimal..2.suboptimal
## 1                                         1
## 2                                         1
## 3                                         2
## 4                                         1
## 5                                         1
## 6                                         2
##   staging.1.IIIA.2.IIIB.3.IIIC.4.IV
## 1                                 3
## 2                                 3
## 3                                 3
## 4                                 3
## 5                                 3
## 6                                 3
##   staging.for.calculation.1.stage.III.2.stage.IV grade
## 1                                              1     3
## 2                                              1     3
```

```
## 3                                       1     3
## 4                                       1     1
## 5                                       1     3
## 6                                       1     3
##   two.tier.grading.system..1..type.I.tumor..2.type.II.tumor
## 1                                                         2
## 2                                                         2
## 3                                                         2
## 4                                                         1
## 5                                                         2
## 6                                                         2
##   histologic.types_1..Serous.papillary.adenocarcinoma_.3.Mucinous_4.Endometrioid
## 1                                                                              1
## 2                                                                              1
## 3                                                                              1
## 4                                                                              1
## 5                                                                              1
## 6                                                                              1
##   recurrence_1..Yes._2...No
## 1                         1
## 2                         2
## 3                         1
## 4                         1
## 5                         2
## 6                         1
##   Status_1...alive.without.disease_2...alive.with.disease_3...missing..4...death
## 1                                                                              4
## 2                                                                              1
## 3                                                                              4
## 4                                                                              4
## 5                                                                              1
## 6                                                                              4
##   status.for.calculation..0.alive..1.death.
## 1                                         1
## 2                                         0
## 3                                         1
## 4                                         1
## 5                                         0
## 6                                         1
##   progression.free.survival.months.
## 1                             21.23
## 2                            186.40
## 3                              8.13
## 4                             91.63
## 5                            116.07
## 6                             16.33
##   overall.survival.months. nTumour nOthers nLymphozyte
## 1                    55.83  201058  154990       29747
## 2                   186.40   80930  111802       16602
## 3                    15.50  343747  118165       38472
## 4                   110.97  175004   64882       15186
## 5                   116.07  687492   15659      165434
## 6                    59.40  336918  167156       46507
```

```
##      cancer        lym    stromal lymPath cancerPath
## 1 0.5211524 0.07710572 0.40174186       1         71
## 2 0.3866070 0.07930866 0.53408429       2         63
## 3 0.6869664 0.07688495 0.23614864       2         65
## 4 0.6860965 0.05953613 0.25436739       1         37
## 5 0.7915080 0.19046380 0.01802817       2         82
## 6 0.6119318 0.08446895 0.30359929       2         61
##   stromalPath S_10year OS_10year type
## 1          29    21.23     55.83    2
## 2          37  120.00+   120.00+    2
## 3          35     8.13     15.50    2
## 4          63    91.63    110.97    1
## 5          18  116.07+   116.07+    2
## 6          39    16.33     59.40    2
##   tumor.types_1..High.Grade.Serous.papillary.adenocarcinoma_2..Low.Grade.Serous.papillary.a
## 1
## 2
## 3
## 4
## 5
## 6
```

The summary statistics of PFS survival:

```
summary(trait$S_10year)

##       time            status
##  Min.   :  0.97   Min.   :0.0000
##  1st Qu.: 10.46   1st Qu.:1.0000
##  Median : 22.00   Median :1.0000
##  Mean   : 34.04   Mean   :0.7802
##  3rd Qu.: 48.69   3rd Qu.:1.0000
##  Max.   :120.00   Max.   :1.0000
```

Now the estimated median follow-up to recurrence can be calculated by the reverse Kaplan-Meier method. We invert the censoring index for death to estimate time to loss of follow up.

```
survfit(Surv(trait$S_10year[,1], trait$S_10year[,2]==0) ~ 1)

## Call: survfit(formula = Surv(trait$S_10year[, 1], trait$S_10year[,
##     2] == 0) ~ 1)
##
##       n  events  median 0.95LCL 0.95UCL
##    91.0    20.0    79.7    65.1   114.6
```

## 2 Distribution

We first examine the distribution of stromal ratio according to clinical parameters:

```
df <- trait
measureLabel <- "Stromal"
measure <- trait$stromal
par(mfrow = c(2, 2), mar = c(4, 4, 3, 1))
```

```
# Debulking
print(paste(measureLabel, ": Debulking"))

## [1] "Stromal : Debulking"

Category = factor(df$debulking, levels = c(1, 2), labels = c("Optimal",
    "Suboptimal"))
PVAL = kruskal.test(measure ~ Category)$p.value
# PVAL = summary(aov(measure ~ Category))[[1]][1,5]
boxplot(measure ~ Category, main = paste0("p = ", signif(PVAL,
    2)), ylab = measureLabel, xlab = "Debulking", varwidth = T)
# Age
print(paste(measureLabel, ": Age"))

## [1] "Stromal : Age"

Category = factor(df$age.for.calculation, levels = 1:2, labels = c("<60",
    ">60"))
PVAL = kruskal.test(measure ~ Category)$p.value
# PVAL = summary(aov(measure ~ Category))[[1]][1,5]
boxplot(measure ~ Category, main = paste0("p = ", signif(PVAL,
    2)), ylab = measureLabel, xlab = "Age", varwidth = T)
# Stage
print(paste(measureLabel, ": Stage"))

## [1] "Stromal : Stage"

Category = factor(df$staging.for.calculation, levels = 1:2, labels = c("3",
    "4"))
PVAL = kruskal.test(measure ~ Category)$p.value
# PVAL = summary(aov(measure ~ Category))[[1]][1,5]
boxplot(measure ~ Category, main = paste0("p = ", signif(PVAL,
    2)), ylab = measureLabel, xlab = "Stage", varwidth = T)
# Histology
print(paste(measureLabel, ": Histology"))

## [1] "Stromal : Histology"

Category = factor(df$tumor.type, levels = c(1, 2, 3, 4), c("HGSC",
    "LGSC", "Mucinous", "Endometrioid"))
PVAL = kruskal.test(measure ~ Category)$p.value
# PVAL = summary(aov(measure ~ Category))[[1]][1,5]
boxplot(measure ~ Category, main = paste0("p = ", signif(PVAL,
    2)), ylab = measureLabel, xlab = "Histology", varwidth = T)
```

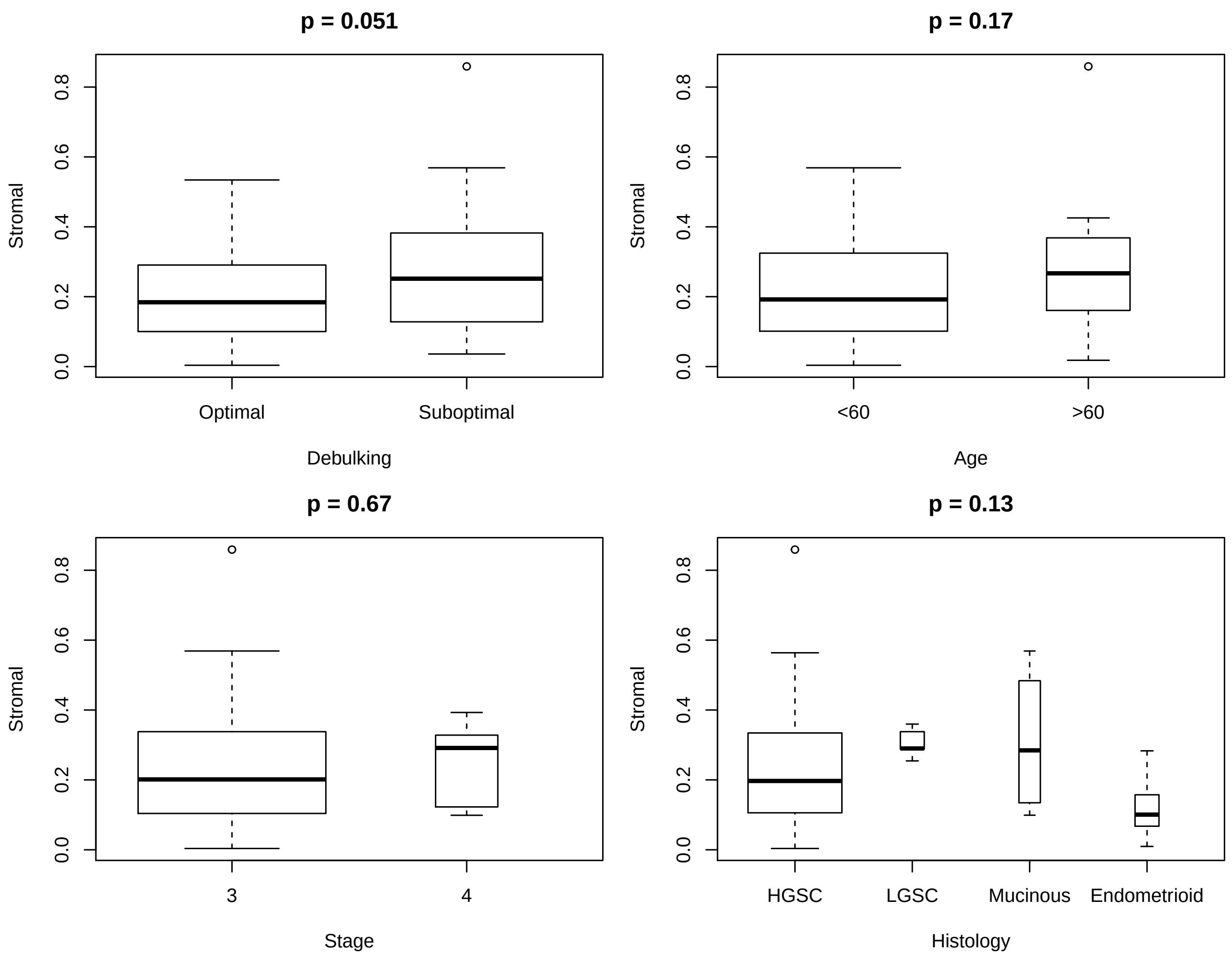


Then lymphocyte ratio:

```
df <- trait
measureLabel <- "Lym"
measure <- trait$lym

par(mfrow = c(2, 2), mar = c(4, 4, 3, 1))
# Debulking
print(paste(measureLabel, ": Debulking"))

## [1] "Lym : Debulking"

Category = factor(df$debulking, levels = c(1, 2), labels = c("Optimal",
    "Suboptimal"))
PVAL = kruskal.test(measure ~ Category)$p.value
# PVAL = summary(aov(measure ~ Category))[[1]][1,5]
boxplot(measure ~ Category, main = paste0("p = ", signif(PVAL,
    2)), ylab = measureLabel, xlab = "Debulking", varwidth = T)
# Age
print(paste(measureLabel, ": Age"))

## [1] "Lym : Age"

Category = factor(df$age.for.calculation, levels = 1:2, labels = c("<60",
    ">60"))
PVAL = kruskal.test(measure ~ Category)$p.value
```

```r
# PVAL = summary(aov(measure ~ Category))[[1]][1,5]
boxplot(measure ~ Category, main = paste0("p = ", signif(PVAL,
    2)), ylab = measureLabel, xlab = "Age", varwidth = T)

# Stage
print(paste(measureLabel, ": Stage"))

## [1] "Lym : Stage"

Category = factor(df$staging.for.calculation, levels = 1:2, labels = c("3",
    "4"))
PVAL = kruskal.test(measure ~ Category)$p.value
# PVAL = summary(aov(measure ~ Category))[[1]][1,5]
boxplot(measure ~ Category, main = paste0("p = ", signif(PVAL,
    2)), ylab = measureLabel, xlab = "Stage", varwidth = T)
# Histology
print(paste(measureLabel, ": Histology"))

## [1] "Lym : Histology"

Category = factor(df$tumor.type, levels = c(1, 2, 3, 4), c("HGSC",
    "LGSC", "Mucinous", "Endometrioid"))
PVAL = kruskal.test(measure ~ Category)$p.value
# PVAL = summary(aov(measure ~ Category))[[1]][1,5]
boxplot(measure ~ Category, main = paste0("p = ", signif(PVAL,
    2)), ylab = measureLabel, xlab = "Histology", varwidth = T)
```

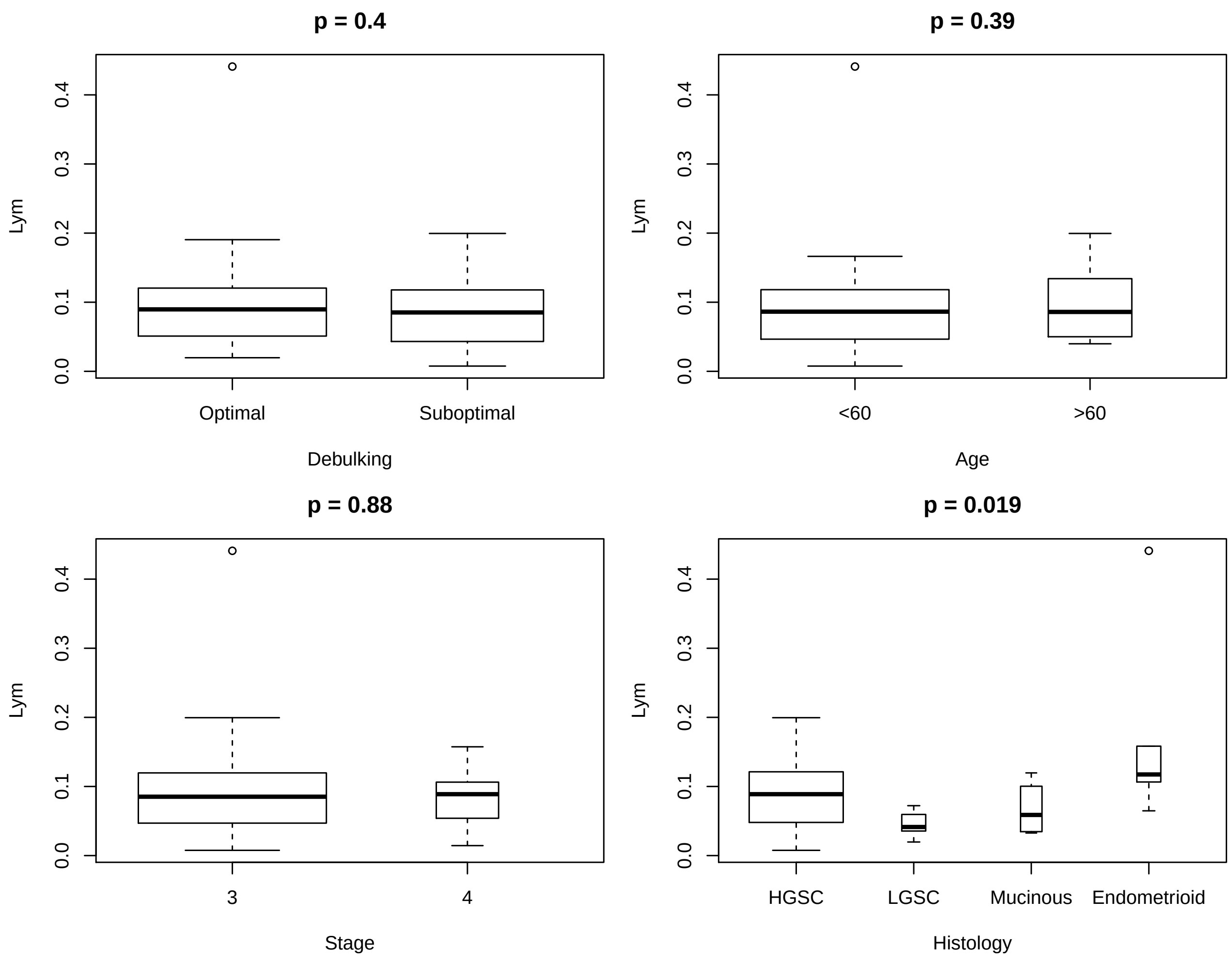


# 3 Prognostic value of the microenvironment

## 3.1 Pathological scores

```
par(mfrow = c(4, 3))
plotSurv(trait$OS_10year, replace.vector(group2(trait$stromalPath),
    1:2, c("Low", "High")), type = "Path H&E score stromal",
    name = "", legendpos = "topright", SurvType = "Overall survival")

## [1] 0.4513003

plotSurv(trait$OS_10year, replace.vector(trait$lymPath, 1:3,
    c("Low", "High", "High")), type = "Path H&E score lym", name = "",
    legendpos = "topright", SurvType = "Overall survival")

## [1] 0.1409078

plotSurv(trait$OS_10year, replace.vector(group2(trait$cancerPath),
    1:2, c("Low", "High")), type = "Path H&E score cancer", name = "",
    legendpos = "topright", SurvType = "Overalle survival")

## [1] 0.4513003

plotSurv(trait$OS_10year, replace.vector(group3(trait$stromalPath),
    1:3, c("Low", "Medium", "High")), type = "Path H&E score stroma",
    name = "", legendpos = "topright", SurvType = "Overall survival")
```

```
## [1] 0.8285458

plotSurv(trait$OS_10year, replace.vector(trait$lymPath, 1:3,
    c("Low", "Medium", "High")), type = "Path H&E score lym",
    name = "", legendpos = "topright", SurvType = "Overall survival")

## [1] 0.2239973

plotSurv(trait$OS_10year, replace.vector(group3(trait$cancerPath),
    1:3, c("Low", "Medium", "High")), type = "Path H&E score cancer",
    name = "", legendpos = "topright", SurvType = "Overall survival")

## [1] 0.6488736

plotSurv(trait$S_10year, replace.vector(group2(trait$stromalPath),
    1:2, c("Low", "High")), type = "Path H&E score stromal",
    name = "", legendpos = "topright", SurvType = "Progression-free survival")

## [1] 0.5448528

plotSurv(trait$S_10year, replace.vector(trait$lymPath, 1:3, c("Low",
    "High", "High")), type = "Path H&E score lym", name = "",
    legendpos = "topright", SurvType = "Progression-free survival")

## [1] 0.1527225

plotSurv(trait$S_10year, replace.vector(group2(trait$cancerPath),
    1:2, c("Low", "High")), type = "Path H&E score cancer", name = "",
    legendpos = "topright", SurvType = "Progression-free survival")

## [1] 0.5448528

plotSurv(trait$S_10year, replace.vector(group3(trait$stromalPath),
    1:3, c("Low", "Medium", "High")), type = "Path H&E score stroma",
    name = "", legendpos = "topright", SurvType = "Progression-free survival")

## [1] 0.7512517

plotSurv(trait$S_10year, replace.vector(trait$lymPath, 1:3, c("Low",
    "Medium", "High")), type = "Path H&E score lym", name = "",
    legendpos = "topright", SurvType = "Progression-free survival")

## [1] 0.3172869

plotSurv(trait$S_10year, replace.vector(group3(trait$cancerPath),
    1:3, c("Low", "Medium", "High")), type = "Path H&E score cancer",
    name = "", legendpos = "topright", SurvType = "Progression-free survival")
```

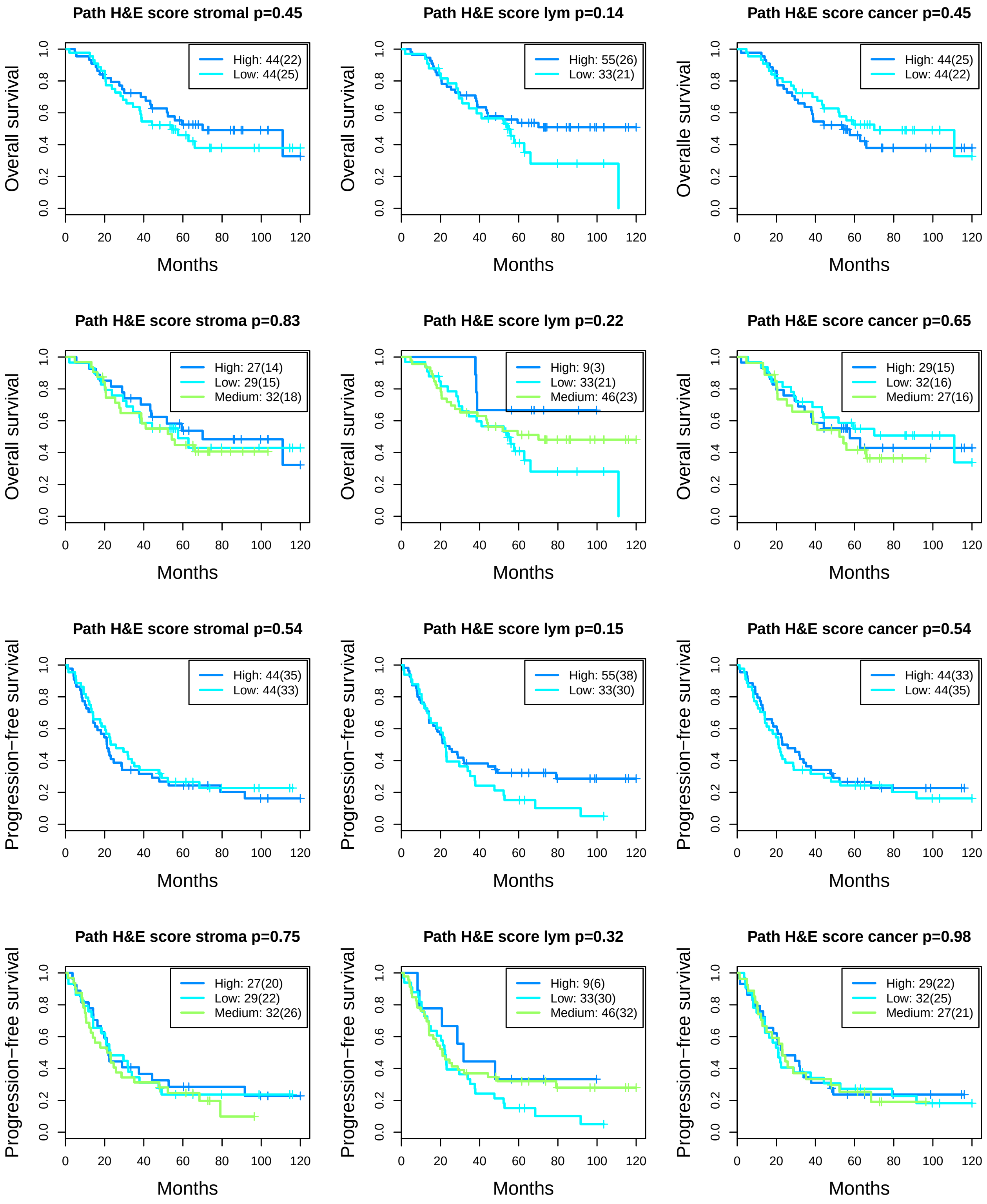


```
## [1] 0.977321
```

## 3.2 Clinical parameters

Only debulking and ge are prognostic.

```
par(mfrow = c(4, 2))
plotSurv(trait$OS_10year, replace.vector(trait$debulking, c(1:2),
```

```
    c("Optimal", "Suboptimal")), type = "Debulking", name = "",
    legendpos = "topright", SurvType = "Overall survival")

## [1] 0.00460321

plotSurv(trait$OS_10year, replace.vector(trait$age.for.calculation,
    1:2, c("<60", ">60")), type = "Age", name = "", legendpos = "topright",
    SurvType = "Overall survival")

## [1] 0.001518249

plotSurv(trait$OS_10year, replace.vector(trait$staging.for.calculation,
    1:2, c("3", "4")), type = "Stage", name = "", legendpos = "topright",
    SurvType = "Overall survival")

## [1] 0.4888366

plotSurv(trait$OS_10year, replace.vector(trait$tumor.types, c(1,
    2, 3, 4), c("HGSC", "LGSC", "Mucinous", "Endometrioid")),
    type = "Histology", name = "", legendpos = "bottomleft",
    SurvType = "Overall survival")

## [1] 0.1219497

plotSurv(trait$S_10year, replace.vector(trait$debulking, c(1:2),
    c("Optimal", "Suboptimal")), type = "Debulking", name = "",
    legendpos = "topright", SurvType = "Progression-free survival")

## [1] 0.002739298

plotSurv(trait$S_10year, replace.vector(trait$age.for.calculation,
    1:2, c("<60", ">60")), type = "Age", name = "", legendpos = "topright",
    SurvType = "Progression-free survival")

## [1] 0.0149717

plotSurv(trait$S_10year, replace.vector(trait$staging.for.calculation,
    1:2, c("III", "IV")), type = "Stage", name = "", legendpos = "topright",
    SurvType = "Progression-free survival")

## [1] 0.5765654

plotSurv(trait$S_10year, replace.vector(trait$tumor.types, c(1,
    2, 3, 4), c("HGSC", "LGSC", "Mucinous", "Endometrioid")),
    type = "Histology", name = "", legendpos = "topright", SurvType = "Progression-free surviv
```

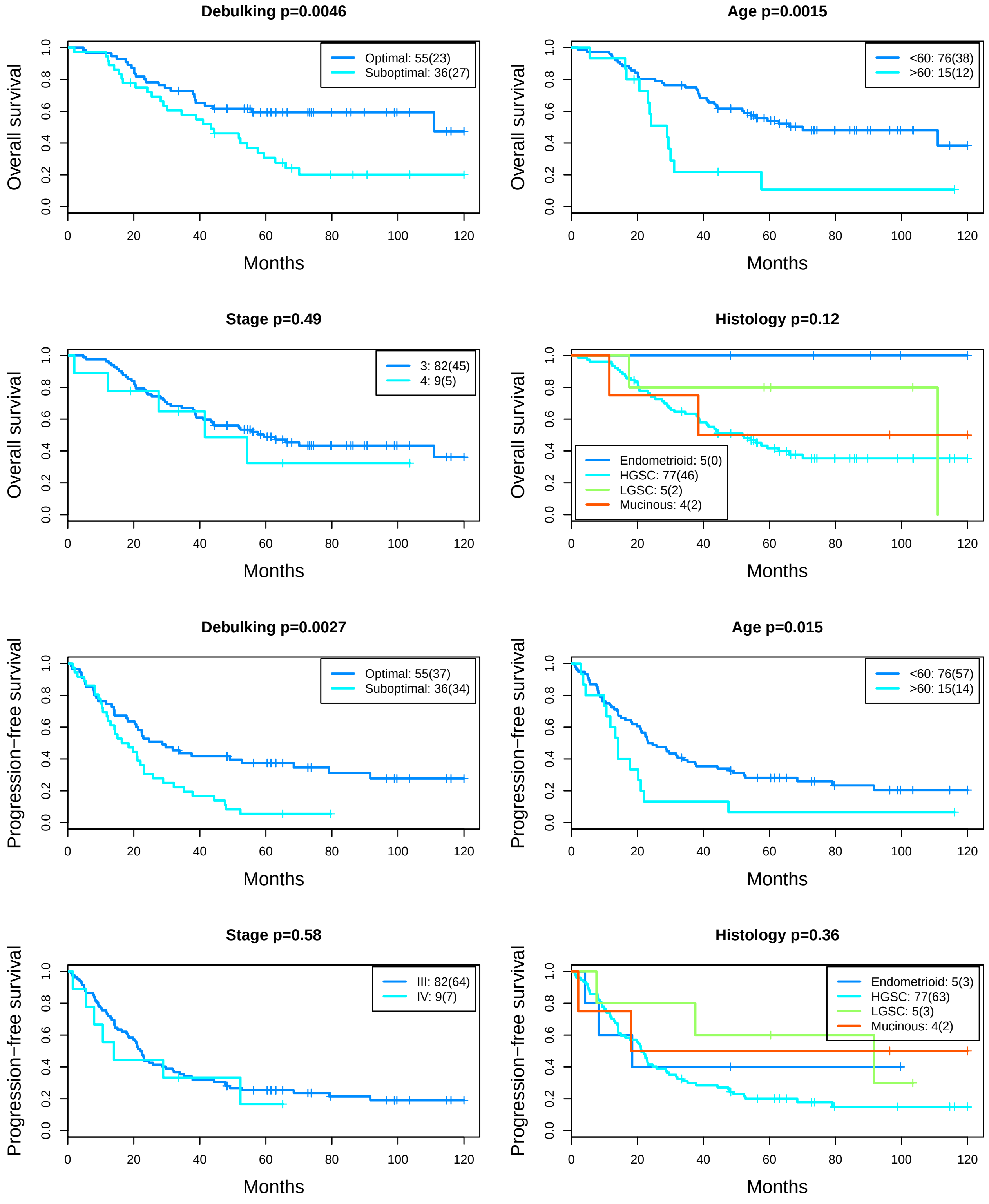


```
## [1] 0.3604023
```

### 3.3 Stromal, lym and cancer on PFS

```
cols <- c("darkblue", "darkred")
par(mfrow = c(2, 3))
plotSurv(trait$S_10year, replace.vector(group2(trait$cancer),
```

```
    1:2, c("Low", "High")), type = "Cancer%", name = "", legendpos = "topright",
    col = cols)

## [1] 0.02344911

plotSurv(trait$S_10year, replace.vector(group2(trait$lym), 1:2,
    c("Low", "High")), type = "Lymphocyte%", name = "", legendpos = "topright",
    col = cols)

## [1] 0.5971327

plotSurv(trait$S_10year, replace.vector(group2(trait$stromal),
    1:2, c("Low", "High")), type = "Stromal%", name = "", legendpos = "topright",
    col = cols)

## [1] 0.01213135

cols <- c("darkblue", "darkred", "black")
plotSurv(trait$S_10year, replace.vector(group3(trait$cancer),
    1:3, c("Low", "Medium", "High")), type = "Cancer%", name = "",
    legendpos = "topright", col = cols)

## [1] 0.04000854

plotSurv(trait$S_10year, replace.vector(group3(trait$lym), 1:3,
    c("Low", "Medium", "High")), type = "Lymphocyte%", name = "",
    legendpos = "topright", col = cols)

## [1] 0.4954263

plotSurv(trait$S_10year, replace.vector(group3(trait$stromal),
    1:3, c("Low", "Medium", "High")), type = "Stromal%", name = "",
    legendpos = "topright", col = cols)
```

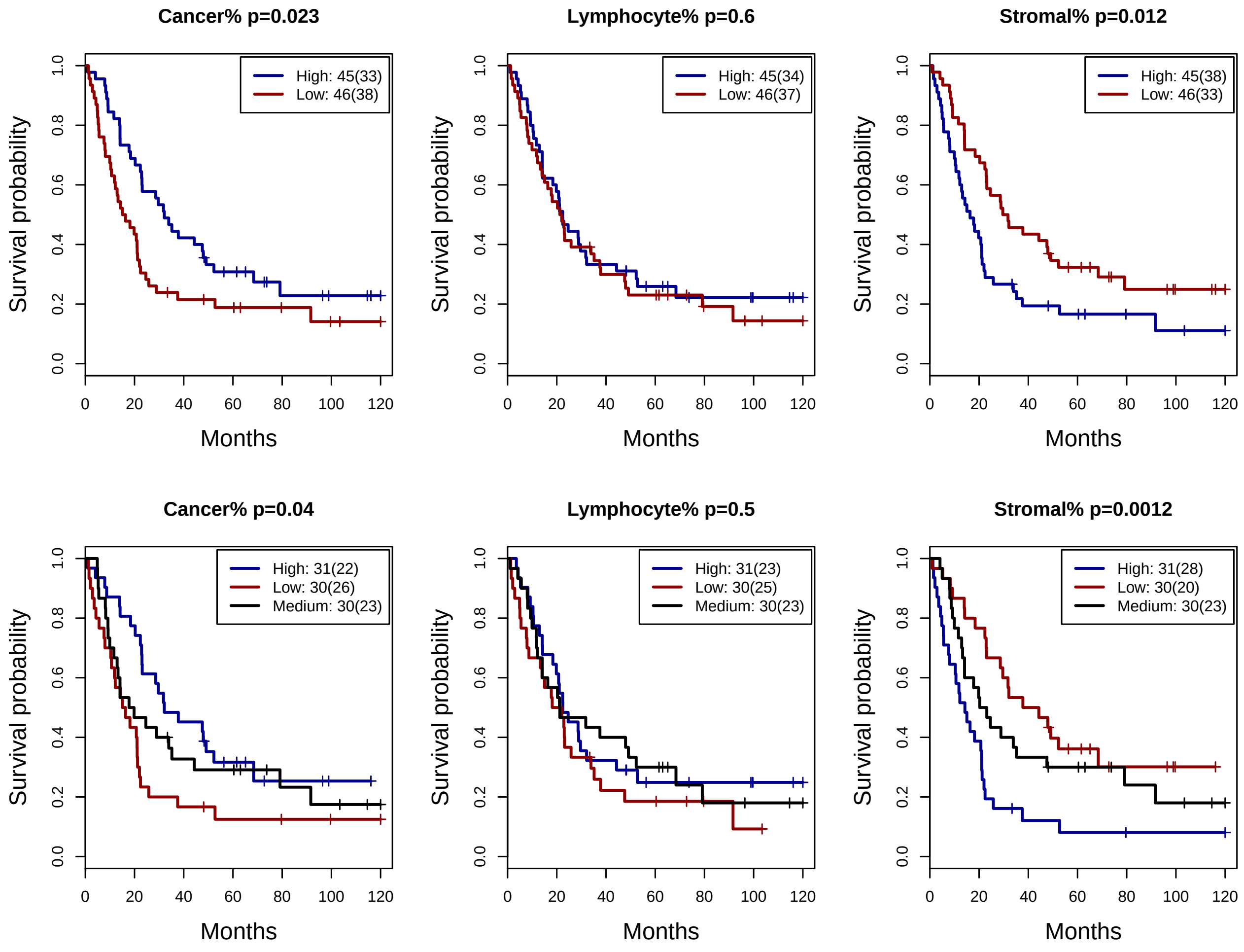


```
## [1] 0.001235623
```

Cox model to include other variables that are also prognostic:

```
summary(coxph(trait$S_10year ~ group2(trait$stromal, th = 0.5) +
    trait$age.for.calculation + trait$debulking))
```

```
## Call:
## coxph(formula = trait$S_10year ~ group2(trait$stromal, th = 0.5) +
##     trait$age.for.calculation + trait$debulking)
##
##   n= 91, number of events= 71
##
##                                   coef  exp(coef)  se(coef)
## group2(trait$stromal, th = 0.5) 0.5586    1.7483    0.2430
## trait$age.for.calculation       0.5868    1.7982    0.3109
## trait$debulking                 0.6240    1.8664    0.2504
##                                     z Pr(>|z|)
## group2(trait$stromal, th = 0.5) 2.299   0.0215 *
## trait$age.for.calculation       1.887   0.0591 .
## trait$debulking                 2.492   0.0127 *
## ---
## Signif. codes:
## 0 '***' 0.001 '**' 0.01 '*' 0.05 '.' 0.1 ' ' 1
##
```

```
##                                    exp(coef) exp(-coef)
## group2(trait$stromal, th = 0.5)      1.748     0.5720
## trait$age.for.calculation            1.798     0.5561
## trait$debulking                      1.866     0.5358
##                                    lower .95 upper .95
## group2(trait$stromal, th = 0.5)     1.0859     2.815
## trait$age.for.calculation           0.9776     3.308
## trait$debulking                     1.1425     3.049
##
## Concordance= 0.622  (se = 0.036 )
## Rsquare= 0.171   (max possible= 0.998 )
## Likelihood ratio test= 17.07  on 3 df,   p=0.0006826
## Wald test            = 17.8  on 3 df,   p=0.0004848
## Score (logrank) test = 18.63  on 3 df,   p=0.0003258
```

### 3.4 Stromal, lym and cancer on OS

```
cols <- c("darkblue", "darkred")
par(mfrow = c(2, 3))
plotSurv(trait$OS_10year, replace.vector(group2(trait$cancer),
    1:2, c("Low", "High")), type = "Cancer%", name = "", legendpos = "topright",
    col = cols)

## [1] 0.00252183

plotSurv(trait$OS_10year, replace.vector(group2(trait$lym, 0.5),
    1:2, c("Low", "High")), type = "Lymphocyte%", name = "",
    legendpos = "topright", col = cols)

## [1] 0.226885

plotSurv(trait$OS_10year, replace.vector(group2(trait$stromal),
    1:2, c("Low", "High")), type = "Stromal%", name = "", legendpos = "topright",
    col = cols)

## [1] 0.0002786323

cols <- c("darkblue", "darkred", "black")
plotSurv(trait$OS_10year, replace.vector(group3(trait$cancer),
    1:3, c("Low", "Medium", "High")), type = "Cancer%", name = "",
    legendpos = "topright", col = cols)

## [1] 0.008473671

plotSurv(trait$OS_10year, replace.vector(group3(trait$lym), 1:3,
    c("Low", "Medium", "High")), type = "Lymphocyte%", name = "",
    legendpos = "topright", col = cols)

## [1] 0.0197518

plotSurv(trait$OS_10year, replace.vector(group3(trait$stromal),
    1:3, c("Low", "Medium", "High")), type = "Stromal%", name = "",
    legendpos = "topright", col = cols)
```

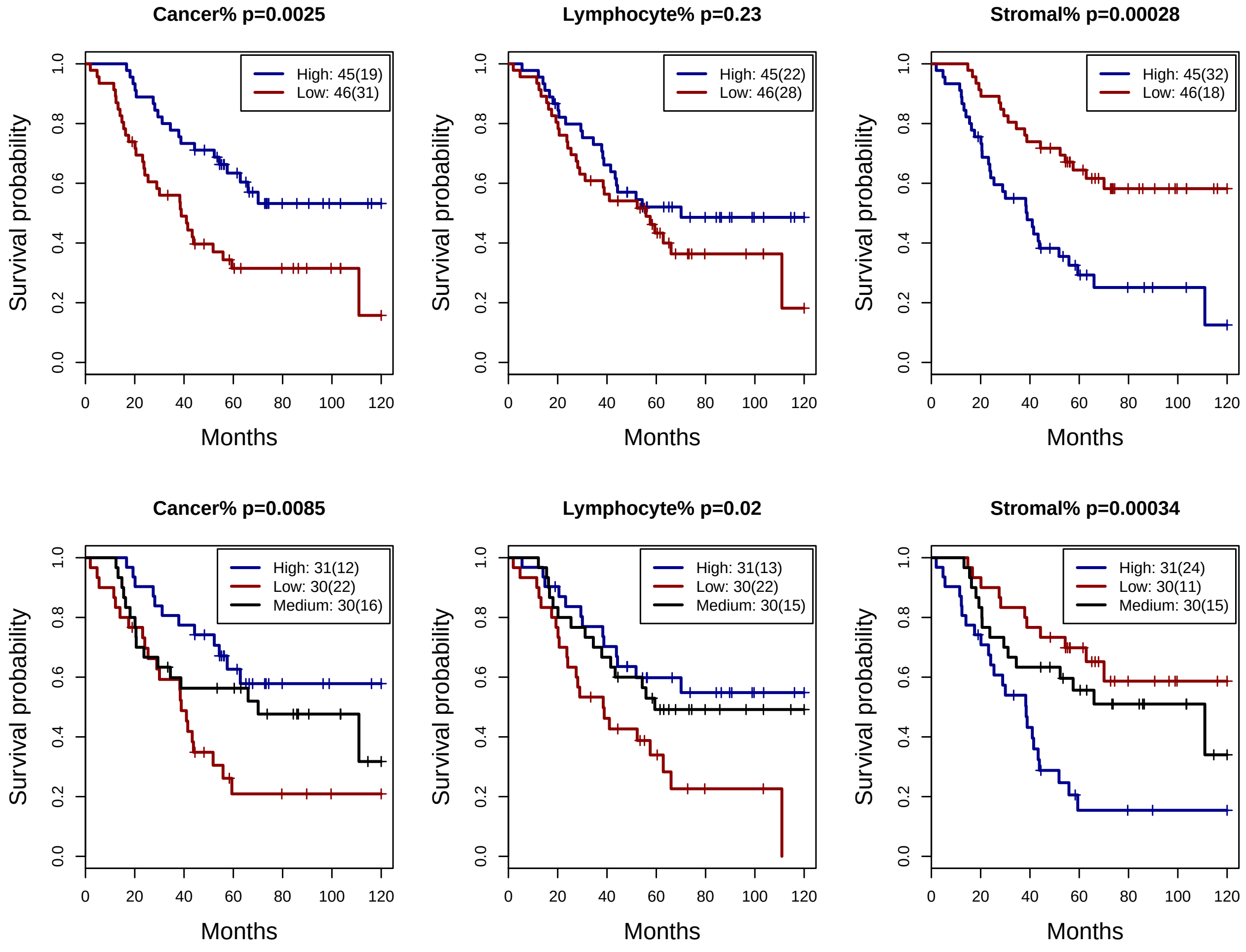


```
## [1] 0.0003446406
```

Cox model to include other variables that are also prognostic:

```
summary(coxph(trait$OS_10year ~ group2(trait$lym, th = 0.33)))

## Call:
## coxph(formula = trait$OS_10year ~ group2(trait$lym, th = 0.33))
##
##   n= 91, number of events= 50
##
##                                  coef exp(coef) se(coef)
## group2(trait$lym, th = 0.33) -0.7722    0.4620   0.2867
##                                   z Pr(>|z|)
## group2(trait$lym, th = 0.33) -2.693  0.00707 **
## ---
## Signif. codes:
## 0 '***' 0.001 '**' 0.01 '*' 0.05 '.' 0.1 ' ' 1
##
##                              exp(coef) exp(-coef) lower .95
## group2(trait$lym, th = 0.33)     0.462      2.164    0.2634
##                              upper .95
## group2(trait$lym, th = 0.33)    0.8104
##
## Concordance= 0.581  (se = 0.034 )
```

```
## Rsquare= 0.073   (max possible= 0.989 )
## Likelihood ratio test= 6.89  on 1 df,   p=0.008677
## Wald test            = 7.25  on 1 df,   p=0.007073
## Score (logrank) test = 7.61  on 1 df,   p=0.0058
```

```
summary(coxph(trait$OS_10year ~ group2(trait$stromal, th = 0.5) +
    trait$age.for.calculation + trait$debulking))
```

```
## Call:
## coxph(formula = trait$OS_10year ~ group2(trait$stromal, th = 0.5) +
##     trait$age.for.calculation + trait$debulking)
##
##   n= 91, number of events= 50
##
##                                   coef exp(coef) se(coef)
## group2(trait$stromal, th = 0.5) 0.9308    2.5366   0.3028
## trait$age.for.calculation       0.8333    2.3010   0.3531
## trait$debulking                 0.5843    1.7938   0.2944
##                                     z Pr(>|z|)
## group2(trait$stromal, th = 0.5) 3.074  0.00211 **
## trait$age.for.calculation       2.360  0.01828 *
## trait$debulking                 1.985  0.04715 *
## ---
## Signif. codes:
## 0 '***' 0.001 '**' 0.01 '*' 0.05 '.' 0.1 ' ' 1
##
##                                 exp(coef) exp(-coef)
## group2(trait$stromal, th = 0.5)     2.537     0.3942
## trait$age.for.calculation           2.301     0.4346
## trait$debulking                     1.794     0.5575
##                                 lower .95 upper .95
## group2(trait$stromal, th = 0.5)     1.401     4.592
## trait$age.for.calculation           1.152     4.597
## trait$debulking                     1.007     3.194
##
## Concordance= 0.69  (se = 0.042 )
## Rsquare= 0.227   (max possible= 0.989 )
## Likelihood ratio test= 23.48  on 3 df,   p=3.212e-05
## Wald test            = 24.12  on 3 df,   p=2.359e-05
## Score (logrank) test = 26.52  on 3 df,   p=7.414e-06
```

```
summary(coxph(trait$OS_10year ~ group2(trait$stromal, th = 0.5) +
    group2(trait$lym, th = 0.66) + trait$age.for.calculation +
    trait$debulking))
```

```
## Call:
## coxph(formula = trait$OS_10year ~ group2(trait$stromal, th = 0.5) +
##     group2(trait$lym, th = 0.66) + trait$age.for.calculation +
##     trait$debulking)
##
##   n= 91, number of events= 50
##
##                                    coef exp(coef) se(coef)
## group2(trait$stromal, th = 0.5)  0.8625    2.3690   0.3068
```

```
## group2(trait$lym, th = 0.66)    -0.3765    0.6863   0.3292
## trait$age.for.calculation        0.8415    2.3198   0.3543
## trait$debulking                  0.5821    1.7898   0.2959
##                                      z Pr(>|z|)
## group2(trait$stromal, th = 0.5)  2.811  0.00493 **
## group2(trait$lym, th = 0.66)    -1.144  0.25276
## trait$age.for.calculation        2.375  0.01753 *
## trait$debulking                  1.967  0.04916 *
## ---
## Signif. codes:
## 0 '***' 0.001 '**' 0.01 '*' 0.05 '.' 0.1 ' ' 1
##
##                                 exp(coef) exp(-coef)
## group2(trait$stromal, th = 0.5)    2.3690     0.4221
## group2(trait$lym, th = 0.66)       0.6863     1.4571
## trait$age.for.calculation          2.3198     0.4311
## trait$debulking                    1.7898     0.5587
##                                 lower .95 upper .95
## group2(trait$stromal, th = 0.5)     1.298     4.322
## group2(trait$lym, th = 0.66)        0.360     1.308
## trait$age.for.calculation           1.159     4.645
## trait$debulking                     1.002     3.197
##
## Concordance= 0.697  (se = 0.043 )
## Rsquare= 0.239   (max possible= 0.989 )
## Likelihood ratio test= 24.85  on 4 df,   p=5.38e-05
## Wald test            = 25.45  on 4 df,   p=4.079e-05
## Score (logrank) test = 27.93  on 4 df,   p=1.29e-05
```

```
summary(coxph(trait$OS_10year ~ group2(trait$lym, th = 0.33) +
    trait$age.for.calculation + trait$debulking))
```

```
## Call:
## coxph(formula = trait$OS_10year ~ group2(trait$lym, th = 0.33) +
##     trait$age.for.calculation + trait$debulking)
##
##   n= 91, number of events= 50
##
##                                 coef exp(coef) se(coef)
## group2(trait$lym, th = 0.33) -0.6440    0.5252   0.2912
## trait$age.for.calculation     0.8473    2.3334   0.3475
## trait$debulking               0.6180    1.8553   0.2933
##                                   z Pr(>|z|)
## group2(trait$lym, th = 0.33) -2.211   0.0270 *
## trait$age.for.calculation     2.439   0.0147 *
## trait$debulking               2.107   0.0351 *
## ---
## Signif. codes:
## 0 '***' 0.001 '**' 0.01 '*' 0.05 '.' 0.1 ' ' 1
##
##                              exp(coef) exp(-coef) lower .95
## group2(trait$lym, th = 0.33)    0.5252     1.9040    0.2968
## trait$age.for.calculation       2.3334     0.4286    1.1809
## trait$debulking                 1.8553     0.5390    1.0441
```

```
##                                upper .95
## group2(trait$lym, th = 0.33)    0.9295
## trait$age.for.calculation       4.6106
## trait$debulking                 3.2968
##
## Concordance= 0.67  (se = 0.041 )
## Rsquare= 0.182   (max possible= 0.989 )
## Likelihood ratio test= 18.29  on 3 df,   p=0.0003838
## Wald test            = 20.38  on 3 df,   p=0.0001416
## Score (logrank) test = 21.84  on 3 df,   p=7.027e-05
```

```
summary(coxph(trait$S_10year ~ group2(trait$lym, th = 0.33) +
    trait$age.for.calculation + trait$debulking))
```

```
## Call:
## coxph(formula = trait$S_10year ~ group2(trait$lym, th = 0.33) +
##     trait$age.for.calculation + trait$debulking)
##
##   n= 91, number of events= 71
##
##                                   coef exp(coef) se(coef)
## group2(trait$lym, th = 0.33) -0.1476    0.8628   0.2548
## trait$age.for.calculation     0.5821    1.7897   0.3085
## trait$debulking               0.6312    1.8799   0.2522
##                                    z Pr(>|z|)
## group2(trait$lym, th = 0.33) -0.579   0.5625
## trait$age.for.calculation     1.887   0.0592 .
## trait$debulking               2.503   0.0123 *
## ---
## Signif. codes:
## 0 '***' 0.001 '**' 0.01 '*' 0.05 '.' 0.1 ' ' 1
##
##                                exp(coef) exp(-coef) lower .95
## group2(trait$lym, th = 0.33)    0.8628     1.1590    0.5237
## trait$age.for.calculation       1.7897     0.5587    0.9777
## trait$debulking                 1.8799     0.5319    1.1467
##                                upper .95
## group2(trait$lym, th = 0.33)     1.422
## trait$age.for.calculation        3.276
## trait$debulking                  3.082
##
## Concordance= 0.607  (se = 0.036 )
## Rsquare= 0.125   (max possible= 0.998 )
## Likelihood ratio test= 12.12  on 3 df,   p=0.006987
## Wald test            = 13.06  on 3 df,   p=0.004511
## Score (logrank) test = 13.63  on 3 df,   p=0.003455
```

```
summary(coxph(trait$OS_10year ~ group2(trait$cancer, th = 0.5) +
    trait$age.for.calculation + trait$debulking))
```

```
## Call:
## coxph(formula = trait$OS_10year ~ group2(trait$cancer, th = 0.5) +
##     trait$age.for.calculation + trait$debulking)
##
```

```
##   n= 91, number of events= 50
##
##                                    coef exp(coef) se(coef)
## group2(trait$cancer, th = 0.5) -0.7812    0.4578   0.2971
## trait$age.for.calculation       0.8729    2.3939   0.3523
## trait$debulking                 0.6098    1.8401   0.2948
##                                      z Pr(>|z|)
## group2(trait$cancer, th = 0.5) -2.629  0.00855 **
## trait$age.for.calculation       2.478  0.01322 *
## trait$debulking                 2.069  0.03858 *
## ---
## Signif. codes:
## 0 '***' 0.001 '**' 0.01 '*' 0.05 '.' 0.1 ' ' 1
##
##                                 exp(coef) exp(-coef)
## group2(trait$cancer, th = 0.5)    0.4578     2.1841
## trait$age.for.calculation         2.3939     0.4177
## trait$debulking                   1.8401     0.5434
##                                 lower .95 upper .95
## group2(trait$cancer, th = 0.5)    0.2558    0.8196
## trait$age.for.calculation         1.2001    4.7751
## trait$debulking                   1.0326    3.2792
##
## Concordance= 0.693  (se = 0.042 )
## Rsquare= 0.204   (max possible= 0.989 )
## Likelihood ratio test= 20.74  on 3 df,   p=0.0001193
## Wald test            = 22  on 3 df,   p=6.519e-05
## Score (logrank) test = 23.86  on 3 df,   p=2.673e-05
```

```
summary(coxph(trait$S_10year ~ group2(trait$cancer, th = 0.5) +
    trait$age.for.calculation + trait$debulking))
```

```
## Call:
## coxph(formula = trait$S_10year ~ group2(trait$cancer, th = 0.5) +
##     trait$age.for.calculation + trait$debulking)
##
##   n= 91, number of events= 71
##
##                                    coef exp(coef) se(coef)
## group2(trait$cancer, th = 0.5) -0.5346    0.5859   0.2425
## trait$age.for.calculation       0.6180    1.8551   0.3118
## trait$debulking                 0.6294    1.8765   0.2516
##                                      z Pr(>|z|)
## group2(trait$cancer, th = 0.5) -2.204   0.0275 *
## trait$age.for.calculation       1.982   0.0475 *
## trait$debulking                 2.502   0.0124 *
## ---
## Signif. codes:
## 0 '***' 0.001 '**' 0.01 '*' 0.05 '.' 0.1 ' ' 1
##
##                                 exp(coef) exp(-coef)
## group2(trait$cancer, th = 0.5)    0.5859     1.7068
## trait$age.for.calculation         1.8551     0.5390
## trait$debulking                   1.8765     0.5329
```

```
##                                       lower .95 upper .95
## group2(trait$cancer, th = 0.5)    0.3642    0.9425
## trait$age.for.calculation         1.0068    3.4184
## trait$debulking                   1.1460    3.0724
##
## Concordance= 0.623  (se = 0.036 )
## Rsquare= 0.167   (max possible= 0.998 )
## Likelihood ratio test= 16.65  on 3 df,   p=0.000833
## Wald test            = 17.54  on 3 df,   p=0.0005462
## Score (logrank) test = 18.33  on 3 df,   p=0.0003767
```

# 4 Combining factors

## 4.1 Clinical and cell ratio

For PFS:

```
par(mfrow = c(2, 3))
plotSurv(trait$S_10year, replace.vector(2 * group2(trait$cancer,
    th = 0.5) - trait$debulking, 0:3, c("Suboptimal, low cancer",
    "Optimal, low cancer", "Suboptimal, high cancer", "Optimal, high cancer")),
    col = c("darkred", "darkred", "darkblue", "darkblue"), lty = c(1,
        2, 1, 2), type = "Debulking+cancer", name = "", legendpos = "topright")

## [1] 0.0007668108

plotSurv(trait$S_10year, replace.vector(2 * group2(trait$lym,
    th = 0.33) - trait$debulking, 0:3, c("Suboptimal, low lym",
    "Optimal, low lym", "Suboptimal, high lym", "Optimal, high lym")),
    col = c("darkred", "darkred", "darkblue", "darkblue"), lty = c(1,
        2, 1, 2), type = "Debulking+lym", name = "", legendpos = "topright")

## [1] 0.009747373

plotSurv(trait$S_10year, replace.vector(2 * group2(trait$stromal,
    th = 0.5) - trait$debulking, 0:3, c("Suboptimal, low stromal",
    "Optimal, low stromal", "Suboptimal, high stromal", "Optimal, high stromal")),
    col = c("darkred", "darkred", "darkblue", "darkblue"), lty = c(1,
        2, 1, 2), type = "Debulking+stromal", name = "", legendpos = "topright")

## [1] 0.000790647

plotSurv(trait$S_10year, replace.vector(2 * group2(trait$cancer,
    th = 0.5) - trait$age.for.calculation, 0:3, c(">60, low cancer",
    "<60, low cancer", ">60,  high cancer", "<60, high cancer")),
    col = c("darkred", "darkred", "darkblue", "darkblue"), lty = c(1,
        2, 1, 2), type = "Age+cancer", name = "", legendpos = "topright")

## [1] 0.0007803699

plotSurv(trait$S_10year, replace.vector(2 * group2(trait$lym,
    th = 0.33) - trait$age.for.calculation, 0:3, c(">60, low lym",
    "<60, low lym", ">60, high lym", "<60, high lym")), col = c("darkred",
    "darkred", "darkblue", "darkblue"), lty = c(1, 2, 1, 2),
    type = "Age+lym", name = "", legendpos = "topright")
```

```
## [1] 0.06266769
```

```
plotSurv(trait$S_10year, replace.vector(2 * group2(trait$stromal,
    th = 0.5) - trait$age.for.calculation, 0:3, c(">60, low stromal",
    "<60, low stromal", ">60, high stromal", "<60, high stromal")),
    col = c("darkred", "darkred", "darkblue", "darkblue"), lty = c(1,
        2, 1, 2), type = "Age+stromal", name = "", legendpos = "topright")
```

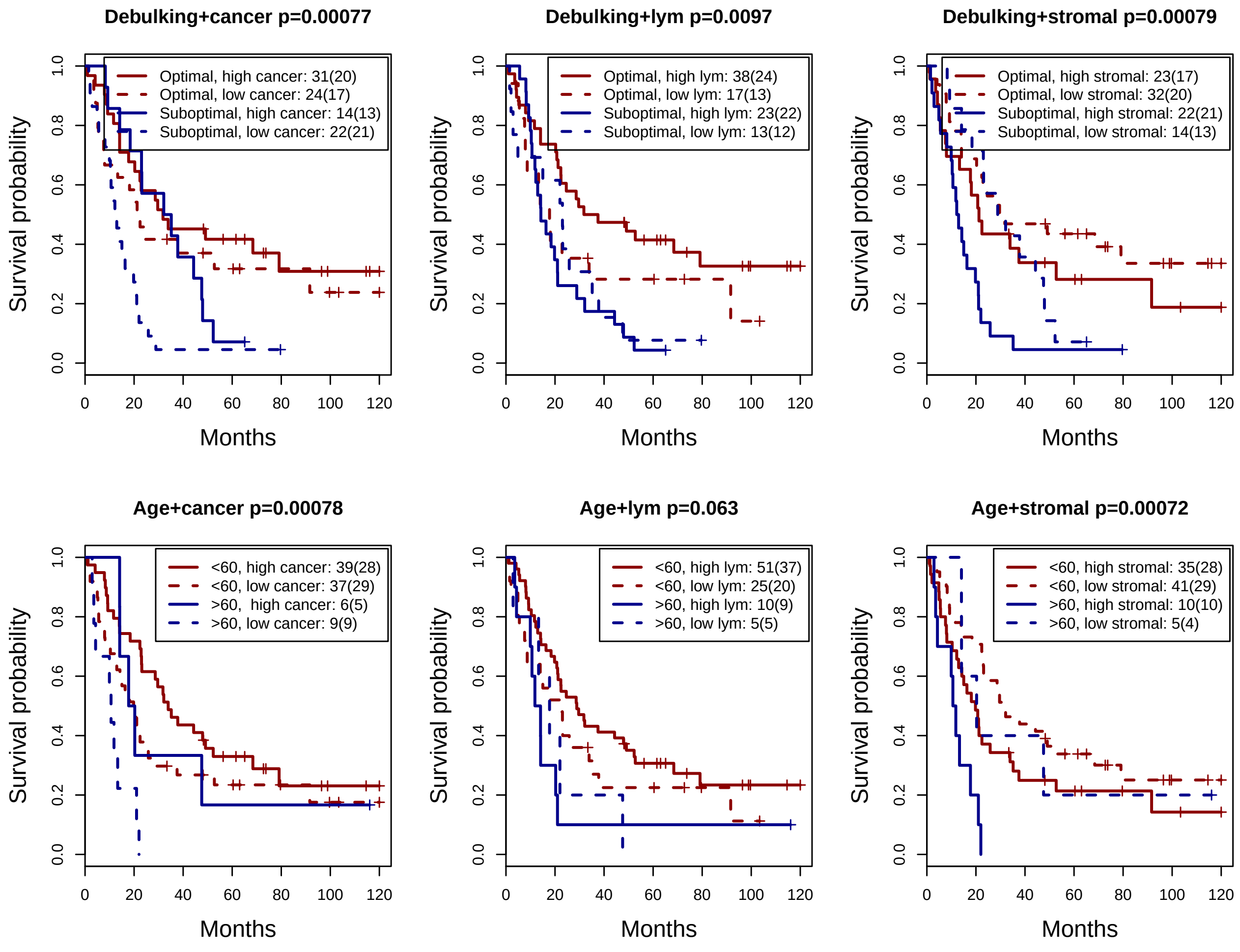


```
## [1] 0.0007238639
```

For OS:

```
par(mfrow = c(2, 3))
plotSurv(trait$OS_10year, replace.vector(2 * group2(trait$cancer,
    th = 0.5) - trait$debulking, 0:3, c("Suboptimal, low cancer",
    "Optimal, low cancer", "Suboptimal, high cancer", "Optimal, high cancer")),
    col = c("darkred", "darkred", "darkblue", "darkblue"), lty = c(1,
        2, 1, 2), type = "Debulking+cancer", name = "", legendpos = "topright")

## [1] 0.001036173

plotSurv(trait$OS_10year, replace.vector(2 * group2(trait$lym,
    th = 0.33) - trait$debulking, 0:3, c("Suboptimal, low lym",
    "Optimal, low lym", "Suboptimal, high lym", "Optimal, high lym")),
    col = c("darkred", "darkred", "darkblue", "darkblue"), lty = c(1,
        2, 1, 2), type = "Debulking+lym", name = "", legendpos = "topright")
```

```
## [1] 0.002637914
```

```
plotSurv(trait$OS_10year, replace.vector(2 * group2(trait$stromal,
    th = 0.5) - trait$debulking, 0:3, c("Suboptimal, low stromal",
    "Optimal, low stromal", "Suboptimal, high stromal", "Optimal, high stromal")),
    col = c("darkred", "darkred", "darkblue", "darkblue"), lty = c(1,
        2, 1, 2), type = "Debulking+stromal", name = "", legendpos = "topright")
```

```
## [1] 0.0001805299
```

```
plotSurv(trait$OS_10year, replace.vector(2 * group2(trait$cancer,
    th = 0.5) - trait$age.for.calculation, 0:3, c(">60, low cancer",
    "<60, low cancer", ">60, high cancer", "<60, high cancer")),
    col = c("darkred", "darkred", "darkblue", "darkblue"), lty = c(1,
        2, 1, 2), type = "Age+cancer", name = "", legendpos = "topright")
```

```
## [1] 0.0002755219
```

```
plotSurv(trait$OS_10year, replace.vector(2 * group2(trait$lym,
    th = 0.33) - trait$age.for.calculation, 0:3, c(">60, low lym",
    "<60, low lym", ">60, high lym", "<60, high lym")), col = c("darkred",
    "darkred", "darkblue", "darkblue"), lty = c(1, 2, 1, 2),
    type = "Age+lym", name = "", legendpos = "topright")
```

```
## [1] 0.0007519826
```

```
plotSurv(trait$OS_10year, replace.vector(2 * group2(trait$stromal,
    th = 0.5) - trait$age.for.calculation, 0:3, c(">60, low stromal",
    "<60, low stromal", ">60, high stromal", "<60, high stromal")),
    col = c("darkred", "darkred", "darkblue", "darkblue"), lty = c(1,
        2, 1, 2), type = "Age+stromal", name = "", legendpos = "topright")
```

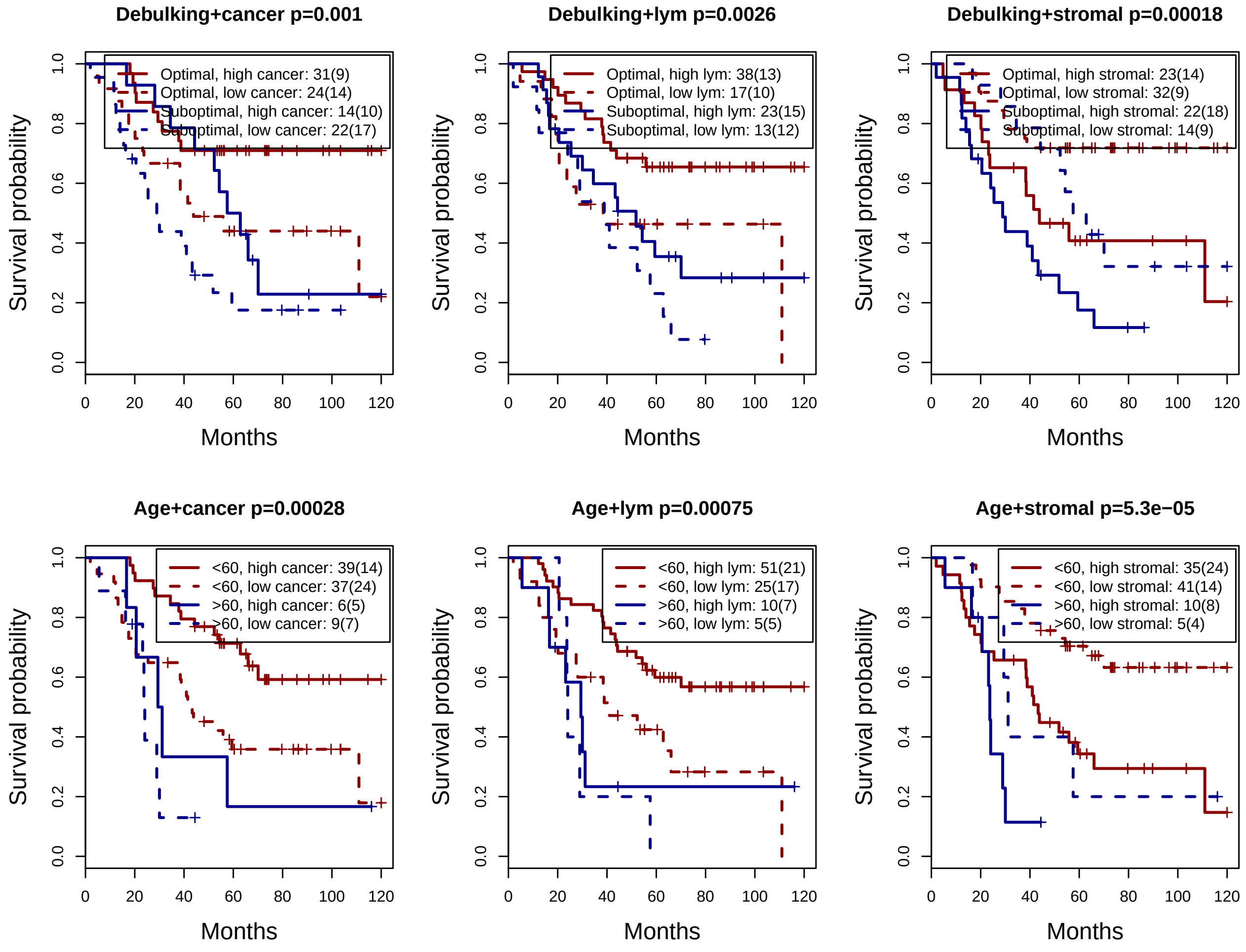


```
## [1] 5.344764e-05
```

## 4.2 Lym and stroma ratio

```
par(mfrow = c(2, 2))
plot(trait$stromal, trait$lym, xlab = "Stroma ratio", ylab = "Lymphocyte ratio",
    main = paste("cor=", signif(cor(trait$stromal, trait$lym,
        use = "complete", method = "spearman"), 2)), pch = 19)
abline(h = quantile(trait$lym, 0.66, na.rm = T), lty = 2)
abline(v = quantile(trait$stromal, 0.66, na.rm = T), lty = 2)
plotSurv(trait$OS_10year, group2(trait$lym, th = 0.66), type = "Lym",
    name = "", legendpos = "topright")

## [1] 0.08443637

plotSurv(trait$OS_10year, group2(trait$stromal, th = 0.66), type = "Stromal",
    name = "", legendpos = "topright")

## [1] 9.976509e-05

# plotSurv(trait$OS_10year, 2*group2(trait$lym, th=.66) -
# group2(trait$stromal, th=.66), type='Lym+Stromal', name='',
# legendpos='topright')
grp <- 2 * (group2(trait$lym, th = 0.66)) - (group2(trait$stromal,
```

```r
    th = 0.66))
grp[grp == 2] <- 0
plotSurv(trait$OS_10year, grp, type = "Lym+Stromal", name = "",
    legendpos = "topright")
```

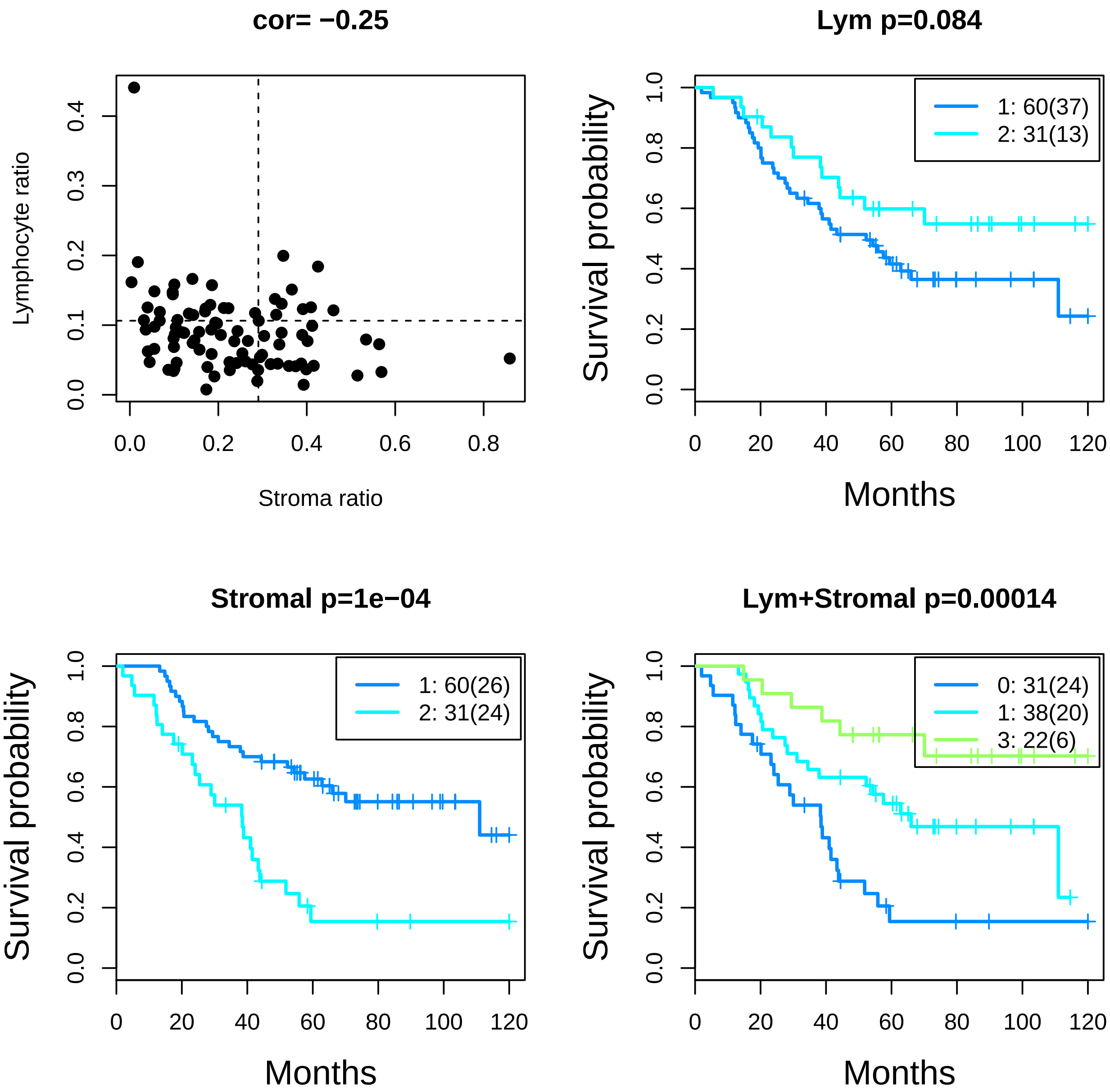


```
## [1] 0.0001449556
```

Cox model to include both variables:

```r
summary(coxph(trait$OS_10year ~ group2(trait$stromal, th = 0.33) +
    group2(trait$lym, th = 0.33)))

## Call:
## coxph(formula = trait$OS_10year ~ group2(trait$stromal, th = 0.33) +
##     group2(trait$lym, th = 0.33))
##
##   n= 91, number of events= 50
```

```
##
##                                      coef exp(coef) se(coef)
## group2(trait$stromal, th = 0.33)  0.7186    2.0515   0.3505
## group2(trait$lym, th = 0.33)     -0.6198    0.5381   0.2931
##                                        z Pr(>|z|)
## group2(trait$stromal, th = 0.33)  2.050   0.0403 *
## group2(trait$lym, th = 0.33)     -2.114   0.0345 *
## ---
## Signif. codes:
## 0 '***' 0.001 '**' 0.01 '*' 0.05 '.' 0.1 ' ' 1
##
##                                  exp(coef) exp(-coef)
## group2(trait$stromal, th = 0.33)    2.0515     0.4874
## group2(trait$lym, th = 0.33)        0.5381     1.8585
##                                  lower .95 upper .95
## group2(trait$stromal, th = 0.33)    1.0322    4.0774
## group2(trait$lym, th = 0.33)        0.3029    0.9558
##
## Concordance= 0.632  (se = 0.041 )
## Rsquare= 0.119   (max possible= 0.989 )
## Likelihood ratio test= 11.57  on 2 df,   p=0.003079
## Wald test            = 10.99  on 2 df,   p=0.004102
## Score (logrank) test = 11.72  on 2 df,   p=0.00285
```

```
summary(coxph(trait$OS_10year[grp != 3] ~ grp[grp != 3]))
```

```
## Call:
## coxph(formula = trait$OS_10year[grp != 3] ~ grp[grp != 3])
##
##   n= 69, number of events= 44
##
##                  coef exp(coef) se(coef)      z Pr(>|z|)
## grp[grp != 3] -0.8072    0.4461   0.3082 -2.619  0.00881 **
## ---
## Signif. codes:
## 0 '***' 0.001 '**' 0.01 '*' 0.05 '.' 0.1 ' ' 1
##
##               exp(coef) exp(-coef) lower .95 upper .95
## grp[grp != 3]    0.4461      2.242    0.2438    0.8161
##
## Concordance= 0.601  (se = 0.04 )
## Rsquare= 0.095   (max possible= 0.991 )
## Likelihood ratio test= 6.86  on 1 df,   p=0.00879
## Wald test            = 6.86  on 1 df,   p=0.008811
## Score (logrank) test = 7.21  on 1 df,   p=0.007251
```

```
summary(coxph(trait$OS_10year ~ grp + +trait$age.for.calculation +
    trait$debulking))
```

```
## Call:
## coxph(formula = trait$OS_10year ~ grp + +trait$age.for.calculation +
##     trait$debulking)
##
##   n= 91, number of events= 50
```

```
##
##                              coef exp(coef) se(coef)      z
## grp                       -0.5167    0.5965   0.1592 -3.246
## trait$age.for.calculation  0.7741    2.1687   0.3579  2.163
## trait$debulking            0.5625    1.7551   0.3011  1.868
##                           Pr(>|z|)
## grp                        0.00117 **
## trait$age.for.calculation  0.03053 *
## trait$debulking            0.06171 .
## ---
## Signif. codes:
## 0 '***' 0.001 '**' 0.01 '*' 0.05 '.' 0.1 ' ' 1
##
##                           exp(coef) exp(-coef) lower .95
## grp                          0.5965     1.6765    0.4366
## trait$age.for.calculation    2.1687     0.4611    1.0754
## trait$debulking              1.7551     0.5698    0.9728
##                           upper .95
## grp                          0.8149
## trait$age.for.calculation    4.3733
## trait$debulking              3.1666
##
## Concordance= 0.705  (se = 0.043 )
## Rsquare= 0.251   (max possible= 0.989 )
## Likelihood ratio test= 26.32  on 3 df,   p=8.17e-06
## Wald test            = 25.45  on 3 df,   p=1.241e-05
## Score (logrank) test = 28.26  on 3 df,   p=3.204e-06
```

```
summary(coxph(trait$S_10year ~ grp + +trait$age.for.calculation +
    trait$debulking))
```

```
## Call:
## coxph(formula = trait$S_10year ~ grp + +trait$age.for.calculation +
##     trait$debulking)
##
##   n= 91, number of events= 71
##
##                              coef exp(coef) se(coef)      z
## grp                       -0.2763    0.7586   0.1228 -2.251
## trait$age.for.calculation  0.5689    1.7664   0.3132  1.817
## trait$debulking            0.5384    1.7133   0.2552  2.110
##                           Pr(>|z|)
## grp                         0.0244 *
## trait$age.for.calculation   0.0693 .
## trait$debulking             0.0348 *
## ---
## Signif. codes:
## 0 '***' 0.001 '**' 0.01 '*' 0.05 '.' 0.1 ' ' 1
##
##                           exp(coef) exp(-coef) lower .95
## grp                          0.7586     1.3183    0.5963
## trait$age.for.calculation    1.7664     0.5661    0.9561
## trait$debulking              1.7133     0.5837    1.0391
##                           upper .95
```

```
## grp                             0.9649
## trait$age.for.calculation       3.2635
## trait$debulking                 2.8251
##
## Concordance= 0.631  (se = 0.037 )
## Rsquare= 0.173   (max possible= 0.998 )
## Likelihood ratio test= 17.3  on 3 df,   p=0.0006135
## Wald test            = 17.77  on 3 df,   p=0.0004912
## Score (logrank) test = 18.63  on 3 df,   p=0.0003253
```

# 5 Only serious cases

```
trait0 <- trait
trait <- trait[trait$histologic.types_1 == 1, ]
par(mfrow = c(4, 3))
plotSurv(trait$S_10year, replace.vector(group2(trait$cancer),
    1:2, c("Low", "High")), type = "Cancer%", name = "", legendpos = "topright",
    col = cols)

## [1] 0.02067901

plotSurv(trait$S_10year, replace.vector(group2(trait$lym), 1:2,
    c("Low", "High")), type = "Lymphocyte%", name = "", legendpos = "topright",
    col = cols)

## [1] 0.8871578

plotSurv(trait$S_10year, replace.vector(group2(trait$stromal),
    1:2, c("Low", "High")), type = "Stromal%", name = "", legendpos = "topright",
    col = cols)

## [1] 0.03082567

cols <- c("darkblue", "black", "darkred")
plotSurv(trait$S_10year, replace.vector(group3(trait$cancer),
    1:3, c("Low", "Medium", "High")), type = "Cancer%", name = "",
    legendpos = "topright", col = cols)

## [1] 0.004312474

plotSurv(trait$S_10year, replace.vector(group3(trait$lym), 1:3,
    c("Low", "Medium", "High")), type = "Lymphocyte%", name = "",
    legendpos = "topright", col = cols)

## [1] 0.4844919

plotSurv(trait$S_10year, replace.vector(group3(trait$stromal),
    1:3, c("Low", "Medium", "High")), type = "Stromal%", name = "",
    legendpos = "topright", col = cols)

## [1] 0.01005391
```

```
plotSurv(trait$OS_10year, replace.vector(group2(trait$cancer),
    1:2, c("Low", "High")), type = "Cancer%", name = "", legendpos = "topright",
    col = cols)

## [1] 0.02533224

plotSurv(trait$OS_10year, replace.vector(group2(trait$lym, 0.5),
    1:2, c("Low", "High")), type = "Lymphocyte%", name = "",
    legendpos = "topright", col = cols)

## [1] 0.4038803

plotSurv(trait$OS_10year, replace.vector(group2(trait$stromal),
    1:2, c("Low", "High")), type = "Stromal%", name = "", legendpos = "topright",
    col = cols)

## [1] 0.008549701

cols <- c("darkblue", "black", "darkred")
plotSurv(trait$OS_10year, replace.vector(group3(trait$cancer),
    1:3, c("Low", "Medium", "High")), type = "Cancer%", name = "",
    legendpos = "topright", col = cols)

## [1] 0.0115186

plotSurv(trait$OS_10year, replace.vector(group3(trait$lym), 1:3,
    c("Low", "Medium", "High")), type = "Lymphocyte%", name = "",
    legendpos = "topright", col = cols)

## [1] 0.1380024

plotSurv(trait$OS_10year, replace.vector(group3(trait$stromal),
    1:3, c("Low", "Medium", "High")), type = "Stromal%", name = "",
    legendpos = "topright", col = cols)
```

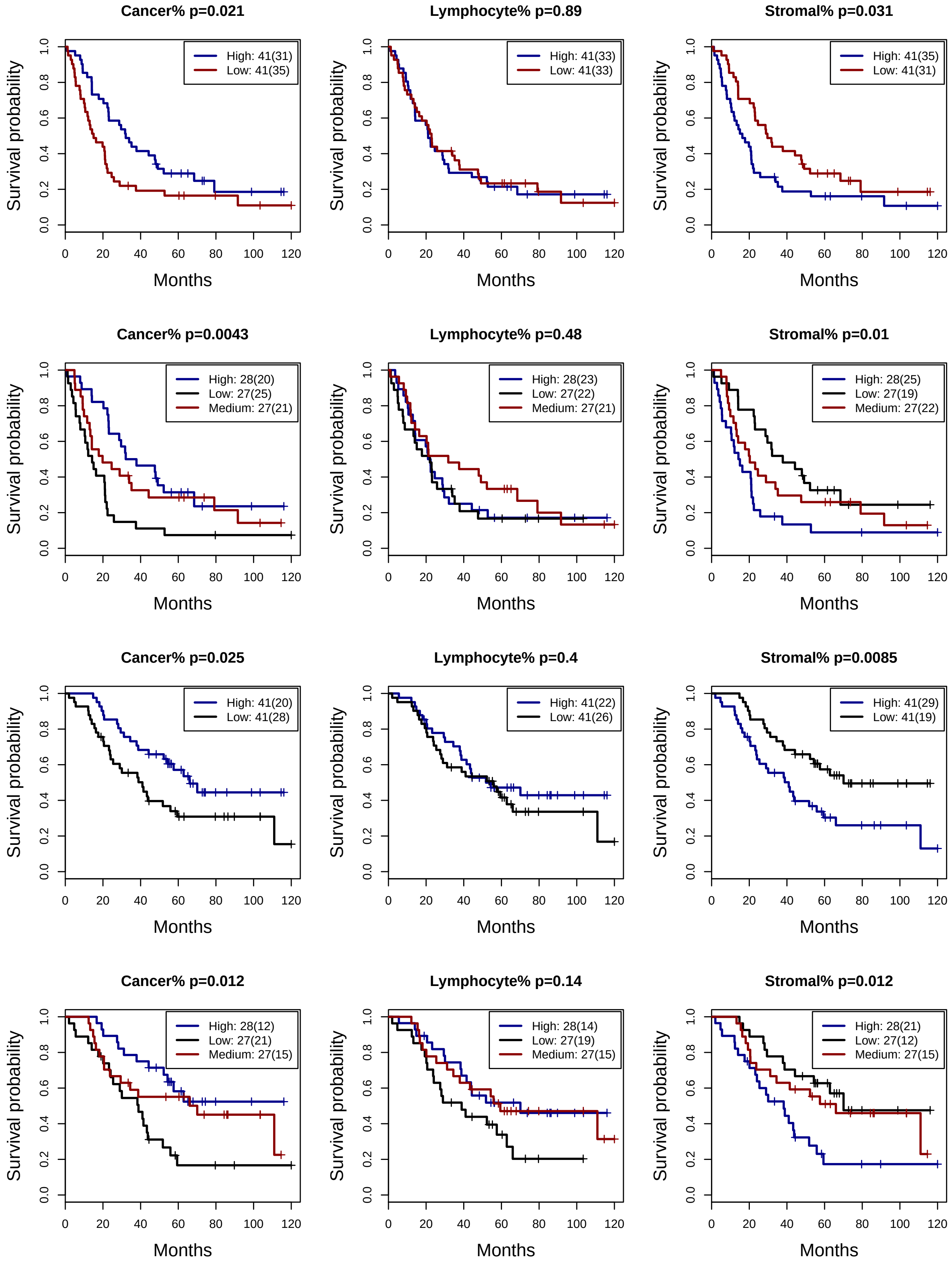


```
## [1] 0.0121178

trait <- trait0
```

# 6 Sampling analysis

## 6.1 Univariate analysis

To test the robustness of Cox model we perform random sampling, first with univariate analysis:

```
set.seed(45)
cl <- replace.vector(group2(trait$stromal), 1:2, c("Low", "High"))
n <- length(cl)
s <- NULL
for (m in seq(1, 0.6, by = -0.01)) {
    resB1 <- sapply(1:1000, function(x) {
        idx <- sample(1:sum(n), size = n * m, replace = FALSE)
        1 - pchisq(survdiff(trait$OS_10year[idx] ~ group2(trait$stromal[idx]))$chisq,
            1)
    })
    s <- c(s, mean(resB1 < 0.05))
}
```

Plot the sampling result:

```
plot(y = seq(1, 0.6, by = -0.01) * 100, x = (s * 100), xlab = "Significance%",
    ylab = "Sampled%", pch = 19)
```

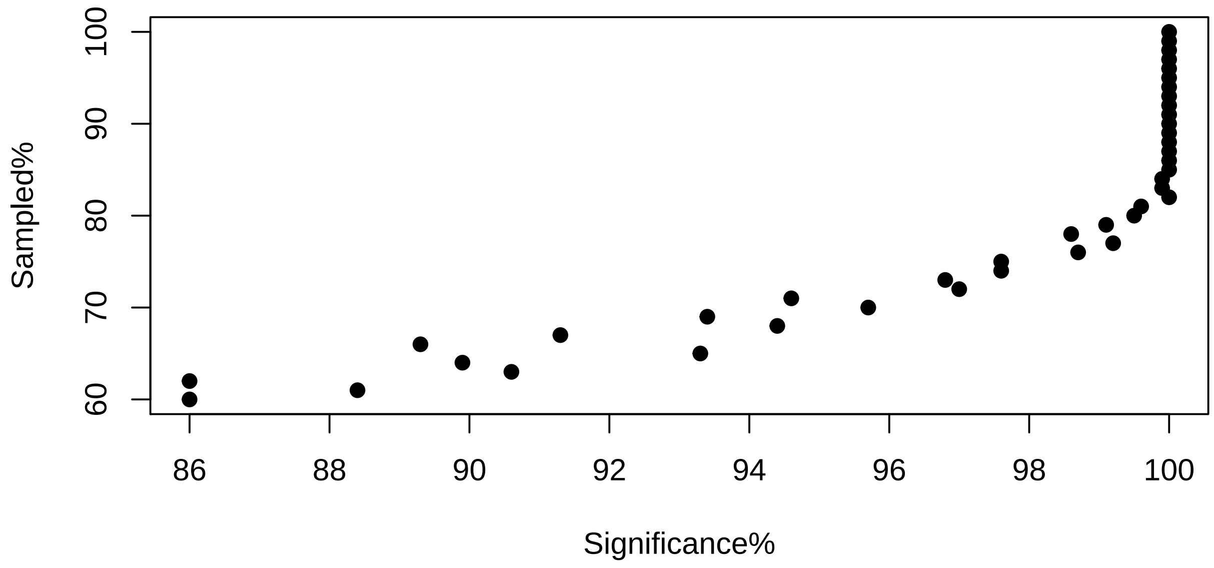


## 6.2 Multivariate analysis

```
set.seed(45)
cl <- replace.vector(group2(trait$stromal), 1:2, c("Low", "High"))
n <- length(cl)
s <- NULL
for (m in seq(1, 0.7, by = -0.01)) {
    resB2 <- sapply(1:1000, function(x) {
```

```
        idx <- sample(1:sum(n), size = n * m, replace = FALSE)
        summary(coxph(trait$OS_10year[idx] ~ group2(trait$stromal[idx]) +
            trait$age.for.calculation[idx] + trait$debulking[idx]))$coef[1,
            5]
    })
    s <- c(s, mean(resB2 < 0.05))
}
```

```
plot(y = seq(1, 0.7, by = -0.01) * 100, x = (s * 100), xlab = "Significance%",
    ylab = "Sampled%", pch = 19)
```

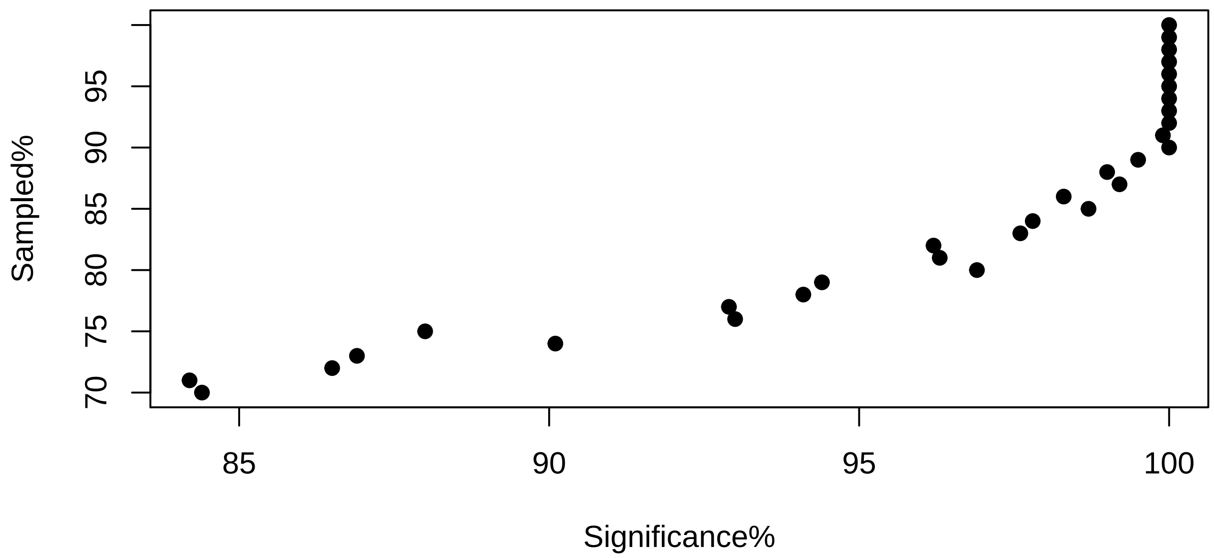

# 7 Session Info

This document was prepared using R package knitr. Funciton knit2pdf("sweave.rnw") was used to compile the sweave file and generate the pdf file.

```
sessionInfo()

## R version 3.2.0 (2015-04-16)
## Platform: x86_64-apple-darwin13.4.0 (64-bit)
## Running under: OS X 10.10.2 (Yosemite)
##
## locale:
## [1] en_GB.UTF-8/en_GB.UTF-8/en_GB.UTF-8/C/en_GB.UTF-8/en_GB.UTF-8
##
## attached base packages:
## [1] stats     graphics  grDevices utils     datasets
## [6] methods   base
##
## other attached packages:
## [1] survival_2.38-3 knitr_1.10.5
##
## loaded via a namespace (and not attached):
## [1] magrittr_1.5  formatR_1.2   tools_3.2.0   splines_3.2.0
## [5] stringi_0.5-5 highr_0.5     stringr_1.0.0 evaluate_0.7
```
